# Tentative Mapping of Transcription-Induced Interchromosomal Interaction using Chimeric EST and mRNA Data

**DOI:** 10.1371/journal.pone.0000254

**Published:** 2007-02-28

**Authors:** Per Unneberg, Jean-Michel Claverie

**Affiliations:** Structural and Genomic Information Laboratory, Centre National de la Recherche Scientifique (CNRS) UPR-2589, Institut de Biologie Structurale et Microbiologie, Marseille, France; Deutsches Krebsforschungszentrum, Germany

## Abstract

Recent studies on chromosome conformation show that chromosomes colocalize in the nucleus, bringing together active genes in transcription factories. This spatial proximity of actively transcribing genes could provide a means for RNA interaction at the transcript level. We have screened public databases for chimeric EST and mRNA sequences with the intent of mapping transcription-induced interchromosomal interactions. We suggest that chimeric transcripts may be the result of close encounters of active genes, either as functional products or “noise” in the transcription process, and that they could be used as probes for chromosome interactions. We have found a total of 5,614 chimeric ESTs and 587 chimeric mRNAs that meet our selection criteria. Due to their higher quality, the mRNA findings are of particular interest and we hope that they may serve as food for thought for specialists in diverse areas of molecular biology.

## Introduction

With the development of increasingly sophisticated large-scale sequencing and microarray techniques, the known transcriptome continues to grow. Even though the idea that one gene produces one protein product has long been abandoned for more complicated models, our understanding of transcription remains incomplete and subject to unexpected findings.

Analyses of transcript databases via bioinformatic approaches have described and uncovered numerous transcript classes. As an example, alternative splicing allows the cell to increase protein diversity – according to current estimates, 75–80% of human genes produce splice variants [1]. Pseudo-messenger RNAs, in the form of expressed pseudogenes or disrupted splice variants with retained introns, have recently been described [2]. Tandem duplication of exons generates non-linear mRNA transcripts [3]. Antisense transcription attests the existence of overlapping gene loci in eukaryotic genomes [4]–[6]. Finally, the never-ending identification of various types of non-protein-coding RNAs (ncRNAs) continues to increase the size and complexity of the transcriptome [7]–[9].

A recent addition to transcript diversity is transcription induced chimerism (TIC) [10], [11]. Here, tandem gene pairs are transcribed into one chimeric transcript, thus generating a fusion protein. The functional role of these proteins remains unclear, but since at least 4–5% tandem gene pairs form chimeric transcripts [11], it is not a singular event. Chimeric microRNA precursor messenger RNA (mRNA) transcripts have also recently been described [12].

In addition to the bioinformatical approaches, new cellular biology techniques are providing new insights in the 3-dimensional and topological properties of the transcription process. The concept of transcription factories – foci for nascent RNA and RNA polymerase II – has been proposed for some time. According to this model, as a gene is activated for transcription, the production of its mRNA takes place in such a transcription factory. Several active genes may occupy the same factory [13]. Lately, it has been shown that even genes from different chromosomes may interact simultaneously in one transcription factory [14], [15]. In fact, the chromosome is now known to be a highly mobile structure, with territories intermingling significantly in the nucleus [16].

It is generally acknowledged that transcript databases in general, and EST databases in particular, contain contaminants of various kinds [17]. Chimeric sequences is one such example. Traditionally, chimeric transcripts have been discarded as artefacts, primarily causing problems in annotation and gene indexing/clustering projects. However, in the light of the radically new findings mentioned previously, it might have been premature to dismiss all aberrant transcripts as artefacts. For instance, *trans*-splicing [18]–[20], the event of joining the exons of two heterologous transcripts, provides a mechanism for the generation of chimeric transcripts. There are few estimates of the frequency of *trans*-splicing events; one study observed ≈0.15% [19], indicating it is a very rare event. Since *trans*-splicing products are generated by the spliceosome machinery, there has been a focus on splicing that occurs at consensus splice sites. However, a study of a rat mRNA, Leukocyte Common Antigen-Related (LAR) tyrosine phosphatase receptor, has indicated the existence of a chimeric transcript with non-consensus splice donor and acceptor sequences [21]. More generally, all kinds of chimeric transcripts could be the product of the normal cell transcriptional process, not discarding the possibility that they may constitute the “noise” of this process, and thus represent *bona fide* biological artefacts, with or without function.

**Figure 1:**
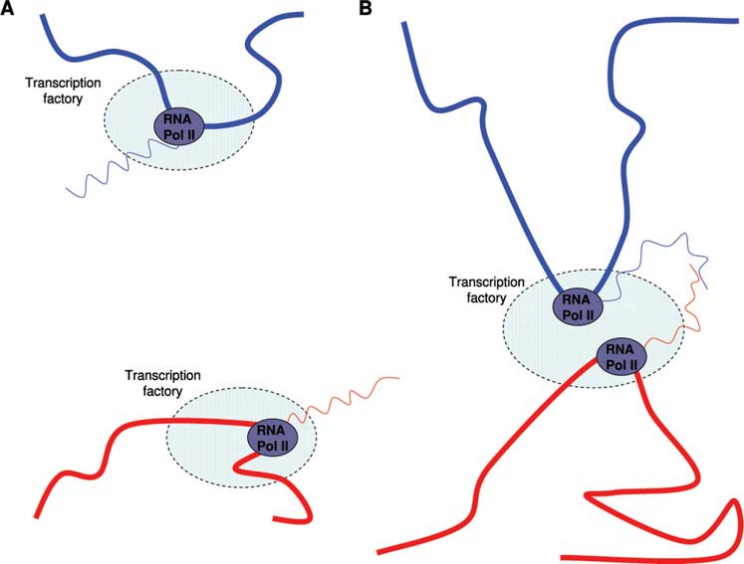
Chimeric mRNA revealing chromsome interaction. Schematic representation of regions of two chromosomes, represented by red and blue thick lines, with accompanying mRNA transcripts in corresponding colors, represented by wavy lines, transcription factories and RNA polymerases. When chromosomes are not in proximity, mRNAs are less likely to interact (A), whereas proximal chromosomes generate a chimeric mRNA, revealing interchromosomal interaction (B).

Chimeric cDNA clones, in that context, might be the unavoidable price to pay for the implementation of local, interchromosomal, gene-coding regulatory processes (Figure 1). Functional or not, the chimeric mRNAs might at least serve as probes for regions of chromosomal interaction taking place in transcription factories. To tentatively map such putative regions of interchromosomal contact, we revisited the specific task of identifying chimeric sequences in transcript databases. Similar procedures have been performed earlier [22], [23], focusing on finding *bona fide* transcripts with exon/exon fusion boundaries. Here, we concentrate on the remaining cases, using the latest human reference genome build.

## Results

### Validation

A recent publication describes ChimerDB, a database that catalogues EST and mRNA fusion sequences in GenBank [24]. In this database only fusion events at exon-exon borders are considered. A natural way to validate our procedure is to compare our chimeric sequences with those found in the database. In the current version (0.8), which is based on NCBI Genome Build version 35, there are 194 EST and 137 mRNA interchromosomal chimeras, respectively. We found 88 (45.1%) of the EST chimeras, and 94 (67.1%) of the mRNA chimeras. The selection criteria used in ChimerDB were alignments of at least 100 bp and 93% identity over the entire query; this difference in imposed criteria turned out to be the main reason for the missed chimeras in the EST case. On the other hand, the missed mRNA chimeras were mainly due to the fact that the ChimerDB chimeras failed to comply with our uniqueness criterion.

### EST mapping results

We mapped 7684642 EST sequences to the human genome reference sequence. 5702 ESTs passed the selection criteria outlined in Methods; 5614 remained after removing the already known fusion sequences found in the validation step. The mean chimeric EST length was 507 bp (SD 131), and in total 2844067 bp were mapped.

### Interaction characteristics

A summary of the partner interactions at the locus level is displayed in Table 1. Despite the fact that EST sequences are the basis for the observations, 1546 (27.8%) of the interactions have an intergenic component (Table 1 **A**). Possible explanations for this are either that the EST library was contaminated with genomic DNA, or that the corresponding intergenic transcripts are not yet annotated or characterized (as were most of them until recently) [8], [25]. The latter case would correspond to our hypothesis of transcription-induced interchromosomal interaction.

**Table 1:** Observed chimeric partner interactions.

	IG	GENE				TOTAL
**IG**	162	1402				1564
**GENE**	1402	4050				5452
**A. IG** and **GENE** interactions, 5614 in total.						
	IG	EXON			INTRON	TOTAL
**IG**	162	474			225	861
**EXON**	474	775			324	1573
**INTRON**	225	324			68	617
**B. IG**, **EXON** and **INTRON** interactions, 2028 in total.						
	IG	CDS	5′UTR	3′UTR	INTRON	TOTAL
IG	162	223	33	185	225	828
CDS	223	195	80	238	181	917
5′UTR	33	80	13	35	27	188
3′UTR	185	238	35	117	94	669
INTRON	225	181	27	94	68	595

**C.** **IG**, **CDS**, **5′UTR**, **3′UTR** and **INTRON** interactions, 1876 in total.

Chimeric fusions have been classified as interactions between sequence classes **5′UTR**, **3′UTR**, **EXON**, **GENE**, **IG**, and **INTRON**. For instance, in subtable **C**, there are 33 **IG** - **5′UTR** interactions, meaning that there are 33 chimeric ESTs with an interaction between an intergenic region and a gene; furthermore, the fusion point is located in the **5′UTR** region of the gene partner. The **GENE** class corresponds to cases where no UTR or CDS information exists for the Ensembl gene in question, or the gene had several transcripts that prevented unabmiguous classification of the fusion point. The **TOTAL** column indicates the total number of times a given class participates in an interaction.

**Figure 2:**
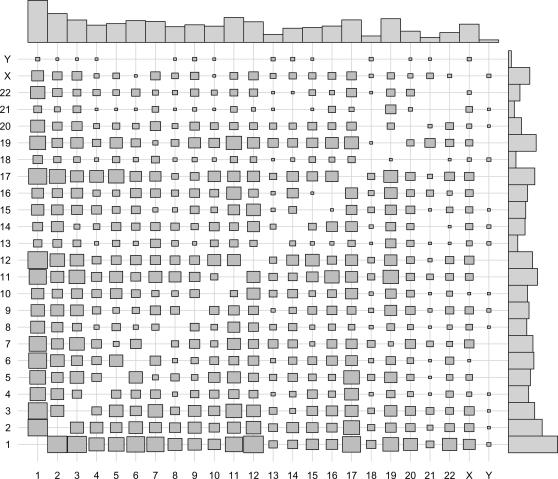
Gene interaction plot. Mosaic plot of gene interactions for 2651 EST chimeras where the direction of the participating partners is the same. The size of each square is proportional to the number of times a fusion event is observed between chromosomes *i* and *j*, for *i*,*j*∈1,2,…,22,*X*,*Y*. The barplots represent known gene densities on each chromosome, according to Ensembl gene counts for all chromosomes.

The 4050 remaining cases (72.3%) represent potential gene–gene interactions. The orientation could be determined for both partners in 3621 chimeras, out of which 2651 consisted of partners that were oriented in the same direction. Figure 2 shows the frequency of chromosome interactions for this subset, where the size of each square is proportional to the number of times a given chromosome–chromosome pair is observed. Plotted above and to the right of the cell frequencies are the gene frequencies for each chromosome. The gene frequencies tell us what to expect if the associations between chromosomes are non-preferential. For instance, since the number of genes is highest on chromosome 1, followed by chromosome 2, one would expect the highest number of observed interactions between these chromosomes; this indeed is the case. The correlation between the observed values and expected values as calculated with gene frequencies is 0.72, confirming the non-preferential nature of the observed associations.

The ESTs with gene partners having the same direction are potential sources of *trans*-splicing observations if the fusion point occurred at exon boundaries for both partners. Exon boundaries could be uniquely defined for both partners in 1852 cases. Accepting a distance of 10 bp from a boundary, we observed only 20 potential *trans*-splicing cases. Consequently, *trans*-splicing is not a main reason for EST chimera generation in this data set.

### EST library distribution

**Figure 3:**
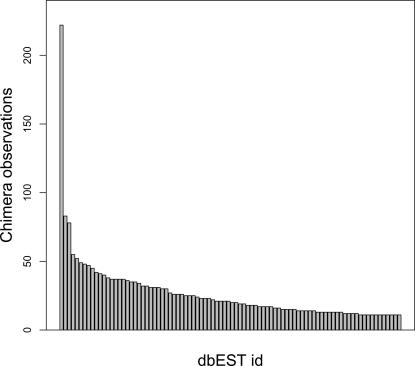
Chimera distributions by EST library. Distribution of chimeric observations grouped by dbEST library id. Only libraries with more than 10 observations are shown.

At the time of analysis, the human EST sequences could be derived from 8618 EST libraries. The 5614 EST sequences that passed the validation criteria originated from 1537 libraries, with 756 libraries contributing one sequence. Figure 3 illustrates a barplot of EST library counts for libraries with more than 10 contributing sequences.

The most commonly observed library, NCI_CGAP_GC6 (dbEST library id 1402), contributes 222 chimeric sequences, followed by libraries Soares_NFL_T_GBC_S1 (library id 1042) and Fetal brain, Stratagene (cat#936206) (library id 2). Table 2 lists the EST libraries with >40 chimeric sequences. Libraries displaying high chimera counts may point to problems in library construction, rather than reflect biologically relevant observations. Contaminated libraries have been analysed by Sorek and Safer (2003), but none of the libraries listed in Table 2 were identified in that study. Nevertheless, it remains possible that the high number of chimeras observed in these libraries is a sign of artefacts in library construction.

**Table 2:** EST libraries with more than 40 observations.

Library id	Library name	Tissue type	Observations	Library size
1402	NCI_CGAP_GC6	pooled germ cell tumors	222	40,001
1042	Soares_NFL_T_GBC_S1	NA	83	68,488
2	Fetal brain, Stratagene (cat#936206)	NA	78	4,222
843	Soares_total_fetus_Nb2HF8_9w	NA	55	27,766
10275	UI-E-EO1	fetal eye	52	7,558
595	NCI_CGAP_GCB1	germinal center B cell	49	52,221
16960	Homo sapiens pancreatic islet	pancreatic islet	48	14,978
589	Soares_NhHMPu_S1	Pooled human melanocyte, fetal heart, and pregnant uterus	47	44,292
452	Soares_fetal_liver_spleen_1NFLS_S1	NA	45	30,928
628	Soares_testis_NHT	NA	42	51,082
1184	Soares_NSF_F8_9W_OT_PA_P_S1	NA	41	26,732

**NA** indicates that no tissue or cell type specification was available for a given dbEST library. **Observations** indicates the number of chimeric observations, and **Library size** indicates the number of EST sequences in a given library.

### mRNA mapping results

Out of 200033 mapped mRNA sequence, 681 passed the selection criteria (Table **S**1), with 587 remaining after removal of known fusion events in the validation phase. In total, 1515785 bp were mapped, with a mean length of 2582 bp (SD 1349).

The random fusion event of two gene-coding mRNA transcripts is likely to alter the reading frame properties of the fusion partners. Even if the fusion occurs at exon-exon boundaries, it is possible that an internal stop codon be introduced in the resulting fusion transcript due to frameshifts. On the other hand, if the fusion occurs between untranslated regions, the open reading frame (ORF) in either partner may remain unaltered. It is often assumed that functional peptides are longer than 100 amino acid residues, although short proteins (e.g. ribosomal proteins) have been shown to be common in the mammalian proteome [26]. 493 (84%) mRNA transcripts have open reading frame lengths ≥300 bp; 265 (45%) have lengths ≥900 bp. Consequently, the majority of mRNA chimeric transcripts code for peptides long enough to be assumed functional.

### Fusion point location in chimera

Apart from reading frame length, the likelihood that a fusion event will affect reading frame characteristics is also dependant on the location of the fusion point. Approximately half (3069 or 54.7%) of the EST chimeric sequences had ORFs that overlapped the fusion point, compared to 27.3% (160 out of 587) for the mRNA chimeras. Due to the imposed selection criteria, the fusion point will be located at least 100 bp from either end of a chimeric sequence. For the shorter EST sequences, it is likely that the fusion point will be distributed halfway through the sequence. However, the mRNAs are full-length sequences, and if the fusion of two sequences occurs in a non-random fashion, the location of the fusion point might also display a non-random pattern.

**Figure 4:**
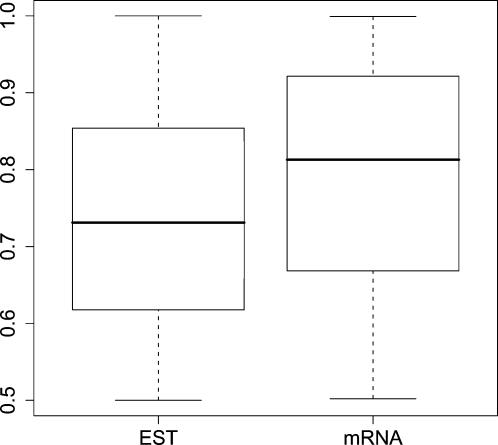
Boxplots of fusion location. Distribution of fusion location for 5614 EST and 587 mRNA chimeras. The fusion location is represented as a fraction *X* of sequence length. Fractions *Y*<0.5 have been transformed to *X* = 1−*Y*.

To investigate this hypothesis, we examined the distribution of the fusion point location. Denote by *X* the fusion point location, and by *L* the sequence length. Since we had imposed that each chimeric partner be at least 100 bp long, *X*∈(100,*L*−100). By letting *Y* = max(*X*,1−*X*)/(*L*−200), *Y* will be uniformly distributed in the range (0.5,1), with a mean value 0.75. Figure 4 shows boxplots of the distribution of *Y* for EST and mRNA sequences. Both plots indicate that the fusion point is randomly distributed along sequence length.

### Interaction examples

The size of each square in the mosaic plot (Figure 2) is proportional to the number of times an EST chimera consists of partners from two given chromosomes. Taking this a step further, a close-up view of each chromosome interaction also provides information about how many times, for instance, a given gene-gene interaction occurs. The simplest way to accomplish this task would be to examine the Ensembl gene ids of the partners in a chimera and count the number of times every pair occurs. However, this procedure would miss unannotated intergenic regions, which nevertheless may be transcribed. Thus, an alternative approach is to determine the mapping (i.e. chromosome start and stop coordinates) for each partner in a chimera. Consequently, all sequences that have chromosome coordinates that overlap are said to have the same mapping.

The majority of EST chimera interactions (4774 out of 5630) are observed only once. However, singleton interactions do not necessarily imply artefactual origin [11], but may reflect low expression levels. There are 12 interactions that are observed ≥5 times (Table 3). Here, except for two cases, all chimeric partners are mapped to genes. Although these are multiple observations, in each case all sequences are from one laboratory, most often also deriving from one EST library. Therefore, it is difficult to ascertain the significance of these findings since there are no independent observations. Due to the smaller mRNA data set, there are only 16 interactions with ≥2 observations (Table 4), of which 5 have an intergenic partner. As pointed out previously, intergenic sequences might indicate that the sequence is contaminated with genomic DNA [17]. However, it is unlikely that a randomly chosen intergenic region would show up more than once in such a small sample that the mRNA data set represents. The interactions IG–GRM7, IG–CDH13, Q96PV3_HUMAN–CK016_HUMAN, SLIT3–MAGED2, and MLL–AFF1, have furthermore been independently observed. Here, it should be remarked that the interactions marked with asterisks are characterized as fusion sequence, although they were not included in ChimerDB. This highlights the difficulty of distinguishing between transcripts that are derived from potential interactions at the transcript level and transcripts that arise from chromosome translocations.

**Table 3:** Most common interactions for EST data.

Left partner		Right partner		Counts			
HUGO gene symbol	Locus	HUGO gene symbol	Locus	Observations	Libids	Labs	Tissuetypes
SAPS2	22q13.33	INTS3	1q21.3	11	2	1	1
RPGF2_HUMAN	4q32.1	IG	7	11	2	1	2
COL6A1	21q22.3	DOT1L	19p13.3	11	1	1	1
FAF1	1p33	DSCR1L1	6p12.3	7	1	1	1
Q96NA9_HUMAN	14q32.2	OGFOD1	16q12.2	6	3	1	2
DDX42	17q23.3	ANKRD44	2q33.1	6	1	1	1
CD40	20q13.12	SEZ6L	22q12.1	5	1	1	1
ATF6	1q23.3	IGF2	11p15.5	5	1	1	1
EIF4G1	3q27.1	RABEP1	17p13.2	5	1	1	1
IG	22	KIAA1279	10q22.1	5	1	1	1
PSMD6	3p14.1	ZNF646	16p11.2	5	1	1	1
SLC5A10	17p11.2	FGG	4q31.3	5	1	1	1

The symbol **IG** indicates that a partner has aligned to a specific intergenic region which is the same for all observations for a given interaction. **Observations** indicates how many times a given interaction has been observed, **Libids** how many different dbEST library ids these observations represent, and similarly for **Labs** and **Tissuetypes**.

**Table 4:** Most common interactions for mRNA data.

Left partner		Right partner		Counts	
HUGO gene symbol	Locus	HUGO gene symbol	Locus	Observations	Labs
IG	19	GRM7	3p26.1	4	2
IG	22	CDH13	16q23.3	3	2
IG	10	NP_057109.2	16p12.2	2	1
Q96PV3_HUMAN	5q33.3	CK016_HUMAN	11p15.4	2	2
KCNJ13	2q37.1	Q96DH5_HUMAN	19p13.3	2	1
IG	12	NIPBL	5p13.2	2	1
TMEFF1	9q31.1	PRKDC	8q11.21	2	1
SLIT3	5q34	MAGED2	Xp11.21	2	2
*MLL	11q23.3	AFF1	4q21.3	2	2
APP	21q21.3	RAB11FIP1	8p12	2	1
HKR2_HUMAN	19q13.43	KIAA1244	6q23.3	2	1
*IG	12	LMBRD1	6q13	2	1
*MLL	11q23.3	Q6AI58_HUMAN	4p12	2	1
*CREB3L2	7q33	FUS	16p11.2	2	1
FOXK2	17q25.3	RAB22A	20q13.32	2	1
C16orf33	16p13.3	HNRPU	1q44	2	1

The symbol **IG** indicates that a partner has aligned to a specific intergenic region which is the same for all observations for a given interaction.

* Corresponds to known fusion events.

## Discussion

We have made a tentative mapping of EST and mRNA sequences to the human genome in the hope of identifying potential gene-gene or locus-locus interactions. Recent findings have shown that interchromosomal interactions upon transcription take place in transcription factories in the nucleus [14]–[16], [27]. As a result, heterologous genes, and consequently transcripts, are colocalized in the nucleus, thereby providing the spatial proximity for possible transcript interaction. Given the recent discoveries concerning transcriptome complexity [3], [8], [10], novel analyses of old transcript data still provide a means for discovering new features in the transcriptome. The focus on canonical splice sites will identify *trans*-splicing events, but any possible alternative mechanism of RNA interaction will go undetected. For this reason, we have applied a search for chimeric sequences without any a priori assumptions about the nature of RNA interactions.

At a first glance, the fact that we observe several interactions more than once in both the EST and mRNA case, would suggest that there is independent evidence corroborating our hypothesis. There are 856 EST sequences that can be grouped into 366 interactions, whereas the majority of interactions are observed once (4758 cases). As shown in Table 3, multiple interaction observations often seem to originate from one or a few libraries. In fact, the 366 interaction groups correspond only to 433 libraries, indicating that multiple observations of an interaction come from the same library. Consequently, there are few independent experiments that validate the multiple observations, thereby questioning their significance.

The large number of rare events may however have other explanations. First, due to the nature of cDNA library construction, EST libraries mostly originate from polyadenylated sequences - the latter may consist of as little as half of all transcribed sequences [25]. Therefore, a large amount of transcripts will be missing from these EST libraries. Second, the choice of mapping parameters has a paramount effect on the number of observed chimeras. EST data are known to contain contaminants and may have sequence error rates as high as 3%. This, in combination with the high repetitive content of the human genome, makes unambiguous mapping difficult, especially for short sequences. Third, EST sequence length limits the number of observed EST chimeras for another reason. Even if a clone is chimeric, the fusion point would, in our settings, have to be located at most 5–600 bp from either clone end (average EST length is 533 bp), and at least 100 bp from either end of the EST sequence. Indeed, the proportion *p* of chimeric sequences is significantly higher in the mRNA data set (*p_mRNA_* = 2.9×10^−3^ vs *p_EST_* = 7.3×10^−4^, z-test, p-value<2.2×10^−16^). A change in filtering settings would affect both data sets, with the difference remaining, and it is likely that this difference can be attributed to sequence length (average mRNA length 1784 bp).

While it is expected that EST sequences are prone to artefacts and contamination problems, the mRNA data set consists of assembled sequences from full-length, cDNA clones, with a higher sequence quality than that of the EST sequences. Still, some 600 intergenic mRNA sequences have been found, of which most are not examples of regular splicing events. The majority have ORFs ≥300 bp; however, only a quarter of ORFs overlap the fusion point, meaning three out of four fusions do not alter reading frame and protein characteristics.

This work presents the first tentative mapping of interchromosomal interactions using EST and mRNA data. The essence of this work is contained in the list of putative locus-locus interactions shown in Table **S**1. This should be considered as a resource on future work on the biological significance of this phenomenon. It is our hope that some of these putative chromosomal interactions might correlate with interesting phenotypes, related to such diverse topics as cytogenetic aberrations in tumours, mutational and recombinational hotspots, and disease-related chromosomal regions. These correlations could be spotted by the trained eye of specialists in their respective fields of research. In addition to the interaction resource, we propose a novel type of transcriptome component that could be derived from the juxtaposition of two regions of different chromosomes. Whether the resulting chimeric transcript solely reports an interaction, or has a specific function, remains to be assessed.

## Materials and Methods

### Data sets

*Homo sapiens* sequence data was taken from GenBank, release 153 (ftp://ftp.ncbi.nih.gov/genbank/). The EST division consisted of 7684642 sequences; 200033 mRNA sequences were obtained from the PRI and HTC divisions. The human genome reference build, release 36.1, was used for the alignments (ftp://ftp.ncbi.nih.gov/genomes/H_sapiens/Assembled_chromosomes). The EST and mRNA data sets were aligned to the human genome reference build, using BLAT [28]. The Ensembl database *homo_sapiens_core_40_36b* was downloaded from ftp://ftp.ensembl.org/pub/current_homo_sapiens/data/mysql/homo_sapiens_core_40_36b. Finally, dbEST report files were downloaded from ftp://ftp.ncbi.nih.gov/repository/dbEST/.

### Selection of chimeric transcripts

Simply put, the selection of chimeric sequences consists of selecting queries (EST or mRNA) that map to loci on two different chromosomes. For each query, all alignments to distinct chromosomes with alignment length ≥100 base pairs (bp) and identity ≥95% were collected and processed. Alignments were sorted according to score, and a transcript was reconstructed by fusing two partner sequences which are defined by the query regions of the two best alignments. A 10 bp overlap between the partner sequences was allowed at the fusion point to account for alignment uncertainties. A query was classified as a chimeric transcript if the number of identities in the two best alignments together constituted ≥95% of the query length.

In addition, the following uniqueness criterion was imposed to make sure that the two best alignments were unambiguous: for a query with three or more alignments, the alignments ranking third or worse were compared with the second best alignment (constituting the “short” partner of the reconstructed transcript). If the ratio of correctly aligned bases between any such alignment and the second alignment was >0.8 **and** the subject regions didn't overlap, then that part was considered to be ambiguously mapped. As a final quality check, the chimera was discarded from further analysis if a restriction recognition site for the enzyme used in library construction was found at the fusion point.

### Classification of interactions

By our selection criteria, the two partners of a chimeric sequence map to loci on two different chromosomes. As a consequence, a chimeric transcript can be viewed as the result of a potential transcriptional interaction between interchromosomal loci. For instance, a potential gene-gene interaction is observed if both partners of a chimeric sequence map to genes. Comparison with the Ensembl database allowed us to classify the fusion point in both partners according to whether they mapped to a gene or an intergenic region, denoted as **GENE** and **IG**, respectively (Table 1 **A**). Moreover, mappings to genes could be classified as **EXON** or **INTRON** if the corresponding Ensembl gene contained transcript(s) with exon/intron information (Table 1 **B**). Finally, if also CDS information existed for the Ensembl gene, for a partner mapping to an exon the fusion point could be further classified as **5′UTR**, **3′UTR** or **CDS** (Table 1 **C**). Alignment of a sequence to gene regions with known transcripts also enabled the inference of sequence orientation.

### Data analysis

We stored EST and mRNA sequences in a MySQL database using the BioSQL schema (http://obda.open-bio.org), adding extra tables for alignment results and dbEST clone library information. The Ensembl database *homo_sapiens_core_40_36b* was installed for the classification of chimera partners and fusion point. Statistical analyses were performed using the software R.

## Supporting Information

Table S1 Mapping results for mRNA sequences. The symbol IG indicates an alignment to an intergenic region.
(1.92 MB DOC)
