# Supplementary material for: Tentative Mapping of Transcription-Induced Interchromosomal Interaction using Chimeric EST and mRNA Data
Source: PLoS One. 2007 Feb 28;2(2):e254. doi: 10.1371/journal.pone.0000254 (PMC1804257; doi:10.1371/journal.pone.0000254)
Supplement: Table S1 — Mapping results for mRNA sequences. The symbol IG indicates an alignment to an intergenic region. (1.92 MB DOC) [file pone.0000254.s001.doc]

|  | |  |  | **Left partner** | | | | | **Right partner** | | | | |
| --- | --- | --- | --- | --- | --- | --- | --- | --- | --- | --- | --- | --- | --- |
| **Accession** | **Length** | | **ORF length** | **Locus** | **Symbol** | **Start** | **End** | **Direction** | **Locus** | **Symbol** | **Start** | **End** | **Direction** |
| AF003522 | 3,162 | | 2,169 | 14q23.1 | RTN1 | 59,132,675 | 59,132,916 | -1 | 6q27 | DLL1 | 170,433,226 | 170,441,227 | 1 |
| AF006515 | 6,331 | | 5,832 | 17p13.1 | CHD3 | 7,732,834 | 7,755,016 | 1 | 9 | IG | 4,164,360 | 4,164,686 | 0 |
| AF025654 | 4,546 | | 1,791 | 1p13.3 | FAM40A | 110,393,602 | 110,393,705 | 1 | 6q15 | RNGTT | 89,376,718 | 89,730,023 | 0 |
| AF032906 | 1,541 | | 960 | 4q21.1 | CCNI | 78,216,037 | 78,216,160 | -1 | 20q13.32 | CTSZ | 57,003,709 | 57,015,697 | 1 |
| AF037331 | 3,823 | | 2,964 | 3q22.1 | P54762-4 | 135,997,155 | 136,461,312 | 0 | 2p23.3 | SNX17 | 27,447,034 | 27,447,665 | 1 |
| AF042345 | 2,013 | | 1,830 | 1p22.1 | EVI5 | 92,862,321 | 93,030,549 | 0 | 10q21.2 | TMEM26 | 62,870,875 | 62,871,068 | -1 |
| AF076463 | 1,341 | | 915 | 4q22.3 | PDLIM5 | 95,758,178 | 95,758,281 | 1 | 1q31.1 | PHOS_HUMAN | 184,679,337 | 184,696,853 | 1 |
| AF112209 | 1,956 | | 1,248 | 17q12 | UNC45B | 30,502,259 | 30,502,380 | -1 | 14q32.2 | EVL | 99,508,077 | 99,680,326 | 0 |
| AF131836 | 1,659 | | 273 | 21q22.2 | DSCAM | 40,877,786 | 40,877,954 | 1 | 9q22.33 | C9orf97 | 99,402,183 | 99,403,663 | 0 |
| AF135593 | 3,495 | | 2,799 | 14q24.2 | RBM25_HUMAN | 72,639,651 | 72,642,455 | 1 | 7p14.1 | VPS41 | 38,731,680 | 38,904,256 | 1 |
| AF225896 | 6,597 | | 5,205 | 12q23.1 | NP_064525.1 | 73,633,606 | 100,638,125 | 0 | 2q35 | TNS1 | 218,376,877 | 218,517,006 | 1 |
| AF241830 | 2,648 | | 2,154 | 20p12.2 | JAG1 | 10,588,896 | 10,589,013 | 1 | 8p11.21 | HOOK3 | 42,871,431 | 42,993,126 | 0 |
| AF307080 | 6,533 | | 3,720 | 13q32.3 | TMTC4 | 100,116,622 | 100,116,787 | 1 | 4q13.1 | LPHN3 | 61,749,970 | 62,620,763 | 0 |
| AF411606 | 1,584 | | 1,470 | 2q14.3 | BIN1 | 127,524,466 | 127,580,989 | 1 | 7 | IG | 150,744,010 | 150,744,117 | 0 |
| AF458052 | 3,389 | | 2,772 | 19 | IG | 32,853,350 | 32,853,645 | 0 | 3p26.1 | GRM7 | 6,877,927 | 7,710,373 | 0 |
| AF458053 | 3,322 | | 2,733 | 19 | IG | 32,853,350 | 32,853,645 | 0 | 3p26.1 | GRM7 | 6,877,927 | 7,710,373 | 0 |
| AF458054 | 3,203 | | 2,718 | 19 | IG | 32,853,350 | 32,853,645 | 0 | 3p26.1 | GRM7 | 6,877,927 | 7,710,373 | 0 |
| AF495726 | 4,013 | | 2,676 | 15q24.2 | C15orf39 | 73,281,290 | 73,290,915 | 1 | 5q31.1 | PCBD2 | 134,287,431 | 134,287,626 | -1 |
| AF497245 | 2,779 | | 846 | 10 | IG | 15,285,503 | 15,285,753 | 0 | 16p12.2 | NP_057109.2 | 21,518,580 | 21,575,897 | 0 |
| AK000234 | 2,935 | | 2,841 | 11q24.3 | PRDM10 | 129,277,469 | 129,317,695 | 1 | 2p11.2 | FABP1 | 88,203,616 | 88,206,897 | 1 |
| AK000306 | 3,319 | | 1,326 | 2q21.2 | NM_153773.1 | 132,728,390 | 132,728,539 | 1 | 9q31.3 | PALM2 | 111,939,332 | 111,972,339 | 1 |
| AK001100 | 2,132 | | 177 | 11 | IG | 121,538,432 | 121,538,640 | 0 | 18q12.1 | DSC3_HUMAN | 26,823,972 | 26,825,894 | 1 |
| AK022287 | 2,766 | | 726 | 11q13.1 | NR_002819.1 | 65,023,322 | 65,023,477 | 1 | 5q35.2 | SFXN1 | 174,838,199 | 174,883,712 | 1 |
| AK022631 | 2,364 | | 2,112 | 2 | IG | 206,567,736 | 206,567,839 | 0 | 1q32.1 | NAV1 | 200,016,277 | 200,039,441 | 1 |
| AK024189 | 2,522 | | 1,857 | 5q31.2 | EIF4EBP3 | 139,873,155 | 139,889,213 | 0 | 19 | IG | 51,996,858 | 51,997,103 | 0 |
| AK024666 | 2,186 | | 1,110 | 2q14.1 | SLC35F5 | 114,188,402 | 114,217,842 | 1 | 1 | IG | 555,649 | 555,822 | 0 |
| AK026660 | 2,351 | | 558 | 1p36.11 | TMEM57 | 25,684,810 | 25,698,825 | 1 | Xq26.3 | NP_660327.2 | 133,731,262 | 133,731,738 | 1 |
| AK026778 | 2,803 | | 318 | 2q22.3 | ZFHX1B | 144,968,302 | 144,970,915 | 1 | 4 | IG | 71,740,124 | 71,740,296 | 0 |
| AK027376 | 1,494 | | 1,065 | 11p15.3 | PARVA | 12,467,056 | 12,467,286 | 1 | 21q22.3 | BACE2 | 41,531,429 | 41,569,648 | 1 |
| AK056053 | 1,727 | | 1,485 | 5q34 | ODZ2 | 167,114,519 | 167,477,910 | 0 | 1q44 | ZNF238 | 242,287,274 | 242,287,397 | 1 |
| AK056989 | 2,108 | | 264 | 8 | IG | 28,972,698 | 28,974,662 | 0 | 1 | IG | 559,473 | 559,617 | 0 |
| AK057254 | 3,037 | | 1,329 | 5q11.2 | NP_075064.1 | 56,574,662 | 56,575,004 | 1 | 16p12.3 | NP_112203.1 | 20,725,347 | 20,768,487 | 0 |
| AK057818 | 1,186 | | 309 | 8p22 | PSD3 | 18,787,904 | 18,788,014 | -1 | 17q11.2 | MYO1D | 27,855,698 | 27,856,775 | 1 |
| AK057997 | 2,290 | | 1,434 | 6q27 | C6orf54 | 168,140,475 | 168,140,646 | 1 | 1q22 | LMNA | 154,351,402 | 154,375,718 | 0 |
| AK074455 | 2,059 | | 1,401 | 5q33.3 | Q96PV3_HUMAN | 159,457,647 | 159,457,770 | 1 | 11p15.4 | CK016_HUMAN | 8,898,199 | 8,911,101 | 0 |
| AK074473 | 2,008 | | 606 | 12p13.31 | CHD4 | 6,580,485 | 6,580,673 | 1 | 20p12.1 | Q9H599_HUMAN | 13,221,023 | 13,229,298 | 1 |
| AK074622 | 1,619 | | 132 | 2q14.2 | CLASP1 | 121,945,422 | 121,945,527 | 1 | 5q21.1 | ST8SIA4 | 100,234,102 | 100,235,614 | 1 |
| AK074709 | 1,174 | | 1,170 | 9q33.3 | OLFML2A | 126,579,258 | 126,606,341 | 1 | 5q35.3 | PDLIM7 | 176,843,003 | 176,843,111 | 1 |
| AK075254 | 2,437 | | 288 | 9q33.1 | C9orf91 | 116,414,613 | 116,416,922 | 1 | 18q21.32 | ZNF532 | 54,776,144 | 54,776,277 | 1 |
| AK090911 | 2,217 | | 1,839 | 17q25.1 | GPR142 | 69,833,947 | 69,860,060 | 0 | 22q11.22 | SUHW2_HUMAN | 21,171,549 | 21,171,789 | -1 |
| AK090930 | 2,131 | | 915 | 6p21.2 | BTBD9_HUMAN | 38,249,689 | 38,656,082 | 0 | 2p24.2 | VSNL1 | 17,700,181 | 17,700,348 | 1 |
| AK091428 | 2,830 | | 1,914 | 6p22.3 | AOF1 | 18,296,002 | 18,330,908 | 1 | 17q21.31 | RUNDC1 | 38,391,719 | 38,391,930 | -1 |
| AK091498 | 3,088 | | 405 | 17p11.2 | SHMT1 | 18,174,722 | 18,177,478 | 1 | 1q21.3 | MLLT11 | 149,307,556 | 149,307,891 | -1 |
| AK092939 | 2,771 | | 630 | 22q12.2 | SMTN | 29,827,862 | 29,830,736 | 1 | 3p21.31 | AMT | 49,429,217 | 49,429,389 | 1 |
| AK092969 | 3,409 | | 498 | 1q32.2 | NP_001034657.1 | 206,053,545 | 206,062,671 | 0 | 19q13.33 | CD37 | 54,535,442 | 54,535,671 | 0 |
| AK097030 | 2,712 | | 2,421 | 16q13 | NP_115582.2 | 55,580,960 | 55,621,421 | 1 | 1p35.2 | PUM1 | 31,220,798 | 31,220,994 | 1 |
| AK097299 | 2,320 | | 1,008 | 12q24.33 | EP400_HUMAN | 131,103,190 | 131,105,307 | 1 | 1p22.3 | LMO4 | 87,583,436 | 87,583,658 | 1 |
| AK098382 | 2,483 | | 264 | 13q21.32 | PCDH9 | 66,554,646 | 66,556,944 | 1 | 1 | IG | 559,434 | 559,617 | 0 |
| AK098390 | 2,367 | | 1,332 | 6p21.33 | G7C_HUMAN | 31,842,045 | 31,853,028 | 1 | 14q32.33 | PPP1R13B | 103,269,842 | 103,270,072 | 1 |
| AK098750 | 2,906 | | 414 | 2 | IG | 101,599,467 | 101,599,635 | 0 | 6p21.1 | VEGF | 43,853,438 | 43,862,154 | 1 |
| AK098753 | 1,593 | | 360 | 2p13.2 | SFXN5 | 73,139,463 | 73,139,613 | 1 | 22q12.1 | Q6ZVA3_HUMAN | 25,395,126 | 25,396,565 | -1 |
| AK122823 | 3,881 | | 426 | 3 | IG | 189,354,027 | 189,354,352 | 0 | 14q32.11 | EML5 | 88,149,818 | 88,155,809 | 1 |
| AK123284 | 2,359 | | 2,271 | 15q21.2 | DMXL2 | 49,560,387 | 49,578,764 | 1 | 1 | IG | 554,570 | 554,674 | 0 |
| AK123444 | 1,738 | | 348 | 1q32.1 | SYT2 | 200,829,521 | 200,831,119 | 1 | 5q31.1 | PCBD2 | 134,291,039 | 134,291,157 | -1 |
| AK123497 | 1,904 | | 426 | 12p11.21 | NP_659410.2 | 31,494,232 | 31,494,336 | 1 | 7q11.23 | BCL7B | 72,588,622 | 72,592,834 | 1 |
| AK123632 | 822 | | 291 | 22 | IG | 35,429,909 | 35,447,440 | 0 | 11q25 | NP_612351.2 | 133,738,966 | 133,739,178 | -1 |
| AK124366 | 2,604 | | 252 | 8 | IG | 119,340,744 | 119,343,227 | 0 | 5q31.1 | PPP2CA | 133,560,681 | 133,560,904 | 1 |
| AK125169 | 2,766 | | 2,466 | 17q23.3 | DDX42 | 59,205,254 | 59,248,944 | 1 | 1q23.2 | DARC | 157,439,525 | 157,439,726 | 1 |
| AK126815 | 2,511 | | 318 | 11 | IG | 126,309,998 | 126,312,256 | 0 | 12p12.1 | SOX5 | 23,787,529 | 23,787,778 | 1 |
| AK126937 | 3,668 | | 3,381 | 5p15.32 | Q6ZT40_HUMAN | 5,475,786 | 5,515,718 | 1 | 19q13.2 | PAFAH1B3 | 47,493,029 | 47,493,187 | 1 |
| AK127251 | 3,826 | | 258 | 18q21.1 | KIAA0427 | 44,345,164 | 44,348,711 | 1 | 16q21 | CNOT1 | 57,111,357 | 57,111,640 | 1 |
| AK127588 | 2,729 | | 309 | 5q13.2 | MAP1B | 71,484,340 | 71,486,823 | 1 | 1q23.1 | ISG20L2 | 154,958,315 | 154,958,562 | 1 |
| AK127824 | 1,737 | | 540 | 4 | IG | 129,951,878 | 129,952,003 | 0 | 18 | IG | 8,614,935 | 8,629,379 | 0 |
| AK128232 | 3,678 | | 570 | 8q11.21 | NP_997387.1 | 49,665,514 | 49,669,734 | 1 | 20q11.23 | NP_542195.1 | 34,952,206 | 34,952,338 | -1 |
| AK128330 | 3,188 | | 2,058 | 17q25.3 | NP_612637.1 | 76,835,010 | 76,874,109 | 0 | 16p13.3 | HAGHL | 719,429 | 719,716 | 1 |
| AK128543 | 4,533 | | 4,416 | 4p12 | Q6AI58_HUMAN | 48,211,983 | 48,261,758 | 1 | 12p13.2 | PRR4 | 10,889,717 | 10,890,986 | 1 |
| AK130258 | 2,832 | | 1,020 | 9p21.2 | IFT74 | 27,002,235 | 27,002,578 | 1 | 2q22.3 | GTDC1 | 144,420,053 | 144,793,907 | 0 |
| AB007865 | 7,527 | | 1,980 | 4p15.31 | KCNIP4 | 21,389,747 | 21,390,735 | -1 | 14q31.3 | FLRT2 | 85,157,474 | 85,164,016 | 1 |
| AB007979 | 5,596 | | 465 | 14q11.2 | NP_060541.3 | 20,612,811 | 20,612,998 | 1 | 1 | IG | 173,550,956 | 173,556,370 | 0 |
| AB013889 | 1,747 | | 1,080 | 19p13.3 | Q96DH5_HUMAN | 3,074,738 | 3,074,917 | -1 | 2q37.1 | KCNJ13 | 233,340,735 | 233,349,454 | 0 |
| AB013891 | 1,747 | | 1,080 | 19p13.3 | Q96DH5_HUMAN | 3,074,738 | 3,074,917 | -1 | 2q37.1 | KCNJ13 | 233,340,735 | 233,349,454 | 0 |
| AB019494 | 7,821 | | 6,795 | 12 | IG | 57,358,568 | 57,358,889 | 0 | 5p13.2 | NIPBL | 37,012,022 | 37,101,050 | 1 |
| AB019602 | 7,308 | | 6,474 | 12 | IG | 57,358,568 | 57,358,889 | 0 | 5p13.2 | NIPBL | 37,012,022 | 37,100,007 | 1 |
| AB023216 | 4,460 | | 3,789 | 2q37.3 | ANKMY1 | 241,145,891 | 241,146,187 | 1 | 11q23.3 | NP_079440.2 | 116,221,338 | 116,474,314 | 0 |
| AB033044 | 5,410 | | 2,499 | X | IG | 73,145,495 | 73,145,603 | 0 | 7q22.3 | ATXN7L4 | 105,032,750 | 105,304,180 | 1 |
| AB058719 | 5,164 | | 3,399 | 5q33.3 | Q6ZP60_HUMAN | 156,743,753 | 156,743,927 | 0 | 4q31.1 | MAML3 | 140,859,092 | 141,294,683 | 0 |
| AY149631 | 4,440 | | 3,189 | 2p21 | THADA | 43,508,740 | 43,676,681 | 1 | 3p25.2 | PPARG | 12,336,071 | 12,336,330 | -1 |
| BC016342 | 1,500 | | 228 | 5 | IG | 151,129,762 | 151,130,024 | 0 | Xq21.1 | Q9H375_HUMAN | 76,968,521 | 76,969,731 | 0 |
| BC017041 | 1,845 | | 282 | 12q23.1 | NA | 97,433,612 | 97,433,730 | 0 | X | IG | 102,047,356 | 102,049,048 | 0 |
| BC025423 | 4,025 | | 3,507 | 8q24.22 | ZNF406 | 135,559,533 | 135,719,111 | 1 | 1q21.3 | S100A1 | 151,871,016 | 151,871,135 | 0 |
| BC042052 | 3,550 | | 321 | 14q32.33 | IGHG1 | 105,401,840 | 105,402,079 | 1 | 8 | IG | 128,767,770 | 128,815,392 | 0 |
| BC048122 | 2,144 | | 528 | 3 | IG | 48,105,820 | 48,105,957 | 0 | 14q12 | COCH | 30,415,136 | 30,428,765 | -1 |
| BC062467 | 1,532 | | 315 | 1p36.32 | NP_997189.1 | 3,652,446 | 3,653,762 | 1 | 7q11.21 | RABGEF1 | 65,881,892 | 65,882,109 | 1 |
| BC071753 | 2,656 | | 1,386 | 11q12.1 | UBE2L6 | 57,084,386 | 57,091,719 | 1 | Xq26.3 | DDX26B | 134,530,928 | 134,544,093 | 1 |
| BC072458 | 1,315 | | 882 | 17p13.1 | PER1 | 7,993,323 | 7,994,889 | 1 | 1 | IG | 172,100,865 | 172,100,971 | 0 |
| X90840 | 6,972 | | 5,070 | 2q37.3 | KIF1A_HUMAN | 241,303,790 | 241,385,904 | 1 | 8q22.3 | NP_056235.3 | 104,513,570 | 104,513,744 | 1 |
| X68485 | 1,942 | | 978 | 7q31.1 | NRCAM | 107,726,309 | 107,726,504 | 1 | 1q32.1 | ADORA1 | 201,363,491 | 201,402,176 | 1 |
| X55330 | 2,150 | | 1,038 | 2p15 | CCT4 | 61,949,084 | 61,949,191 | 1 | 4q34.3 | AGA | 178,588,918 | 178,600,585 | 1 |
| X83961 | 1,672 | | 1,140 | 8q11.21 | PRKDC | 49,019,589 | 49,019,738 | -1 | 9q31.1 | TMEFF1 | 102,275,634 | 102,379,058 | 1 |
| X82068 | 3,128 | | 2,682 | Xq25 | GRIA3 | 122,145,839 | 122,450,291 | 1 | 20q11.22 | PHF20 | 34,000,591 | 34,000,708 | 1 |
| Z46606 | 5,457 | | 3,027 | 13q22.2 | LMO7 | 75,321,310 | 75,321,427 | -1 | 3q24 | SMARCA3 | 150,230,604 | 150,287,007 | 1 |
| AL080107 | 1,583 | | 177 | 4q22.3 | PDLIM5 | 95,709,342 | 95,709,552 | 1 | 2q24.2 | IFIH1 | 162,871,851 | 162,873,215 | 1 |
| AL162082 | 3,442 | | 792 | 17p11.2 | USP22 | 20,844,853 | 20,859,711 | 1 | 12q13.3 | PRIM1 | 55,413,211 | 55,413,774 | 1 |
| AL832587 | 5,304 | | 2,154 | 19p13.11 | SFR14_HUMAN | 18,964,732 | 19,005,348 | 0 | 7 | IG | 150,785,716 | 150,786,262 | 0 |
| BX648932 | 6,681 | | 321 | 2p25.2 | Q96NM1_HUMAN | 6,030,653 | 6,037,216 | 1 | 17 | IG | 72,181,338 | 72,181,794 | 0 |
| X69208 | 2,222 | | 1,875 | 9p22.3 | BNC2 | 16,624,212 | 16,624,399 | -1 | Xq21.1 | ATP7A | 77,052,855 | 77,151,416 | 0 |
| X84075 | 4,575 | | 3,822 | 11p11.2 | MYBPC3 | 47,309,527 | 47,330,798 | 1 | 5 | IG | 99,414,794 | 99,415,071 | 0 |
| Z30425 | 1,450 | | 1,044 | X | IG | 72,958,725 | 72,958,835 | 0 | 1q23.3 | NR1I3 | 159,466,083 | 159,474,585 | 0 |
| X83973 | 2,847 | | 2,700 | 9q34.13 | TTF1 | 134,241,193 | 134,268,037 | 1 | 20q13.13 | STAU1 | 47,173,078 | 47,174,360 | 1 |
| Z75330 | 4,337 | | 3,774 | 17q23.2 | SPT4H_HUMAN | 53,835,076 | 53,835,227 | -1 | 3q22.3 | STAG1 | 137,539,627 | 137,953,899 | 0 |
| X92493 | 2,764 | | 1,620 | Xp22.12 | CNKSR2 | 21,582,477 | 21,582,586 | 1 | 9q21.11 | PIP5K1B | 70,510,436 | 70,813,911 | 0 |
| U09366 | 2,643 | | 1,962 | 16p12.3 | GP2_HUMAN | 20,229,589 | 20,229,938 | -1 | 20p11.23 | ZNF133 | 18,226,628 | 18,245,631 | 1 |
| U09510 | 2,462 | | 2,418 | 14q11.2 | CHD8 | 20,950,912 | 20,952,328 | -1 | 7p14.3 | GARS | 30,600,979 | 30,640,041 | 1 |
| U19878 | 1,672 | | 1,140 | 8q11.21 | PRKDC | 49,019,589 | 49,019,738 | -1 | 9q31.1 | TMEFF1 | 102,275,634 | 102,379,058 | 1 |
| U19969 | 5,126 | | 3,462 | 18p11.22 | RAB31 | 9,712,036 | 9,712,157 | 1 | 10p11.22 | TCF8 | 31,648,186 | 31,857,853 | 0 |
| U28282 | 2,468 | | 1,077 | 2q21.1 | PTPN18 | 130,848,372 | 130,848,625 | -1 | Xp11.21 | KLF8_HUMAN | 56,276,057 | 56,328,379 | 1 |
| U29171 | 1,911 | | 1,245 | 4q31.3 | TRIM2 | 154,417,826 | 154,417,939 | -1 | 17q25.3 | CSNK1D | 77,795,461 | 77,824,606 | 0 |
| U48252 | 1,668 | | 1,371 | 3q27.1 | DVL3 | 185,366,607 | 185,371,352 | 1 | 9q34.11 | FREQ | 132,036,508 | 132,036,656 | -1 |
| U58032 | 1,602 | | 1,488 | 21q22.13 | DYRK1A | 37,731,183 | 37,731,288 | -1 | Xq28 | MTMR1_HUMAN | 149,646,351 | 149,674,992 | 1 |
| U59288 | 3,485 | | 2,139 | 22 | IG | 25,400,569 | 25,400,719 | 0 | 16q23.3 | CDH13 | 81,218,134 | 82,387,253 | 0 |
| U59289 | 3,926 | | 2,139 | 22 | IG | 25,400,569 | 25,400,719 | 0 | 16q23.3 | CDH13 | 81,218,134 | 82,387,702 | 0 |
| U60975 | 6,981 | | 6,837 | 17q21.33 | SPOP | 45,032,412 | 45,032,536 | 1 | 11q23.3 | SORL1 | 120,828,180 | 121,005,622 | 1 |
| U62325 | 2,912 | | 2,190 | 2 | IG | 218,367,976 | 218,368,094 | 0 | 4p14 | APBB2 | 40,512,164 | 40,710,997 | 0 |
| U64876 | 1,791 | | 1,440 | 5q32 | HG2A_HUMAN | 149,772,385 | 149,772,512 | -1 | 9q33.3 | NR6A1 | 126,324,660 | 126,573,294 | 0 |
| U68727 | 3,439 | | 1,308 | 21q22.3 | PKNOX1 | 43,297,501 | 43,325,103 | 1 | Xp22.2 | GPM6B | 13,733,983 | 13,734,202 | -1 |
| U75651 | 3,403 | | 2,148 | 1q32.1 | NFASC_HUMAN | 203,245,397 | 203,245,545 | 1 | 3q27.1 | DVL3 | 185,355,870 | 185,372,098 | 1 |
| U79252 | 1,600 | | 474 | 1q42.12 | ENAH | 223,860,775 | 223,860,894 | 1 | 22q13.1 | NP_061881.2 | 38,242,621 | 38,244,083 | 1 |
| U92458 | 4,312 | | 2,745 | 19 | IG | 32,853,350 | 32,853,645 | 0 | 3p26.1 | GRM7 | 6,877,927 | 7,758,198 | 0 |
| M95178 | 3,081 | | 2,676 | 14q24.1 | ACTN1 | 68,411,289 | 68,515,637 | 1 | 7q22.1 | SERPINE1 | 100,568,993 | 100,569,246 | 1 |
| M12674 | 2,092 | | 1,785 | 7p15.3 | SP4 | 21,519,279 | 21,519,391 | 1 | 6q25.1 | ESR1 | 152,170,568 | 152,461,806 | 0 |
| L20969 | 3,829 | | 2,427 | 6q25.1 | NA | 150,706,739 | 150,706,981 | -1 | 5q11.2 | PDE4D | 58,305,272 | 59,225,378 | 0 |
| M73547 | 3,178 | | 636 | 5q22.2 | REEP5 | 112,239,981 | 112,285,850 | 0 | 1p36.23 | RERE | 8,418,346 | 8,418,455 | -1 |
| M89907 | 2,730 | | 2,304 | 4 | IG | 142,619,182 | 142,619,513 | 0 | Xq25 | SMARCA1 | 128,442,754 | 128,485,122 | 1 |
| D16476 | 1,181 | | 138 | X | IG | 144,686,585 | 144,687,649 | 0 | 14q11.2 | TRDV2 | 21,453,393 | 21,453,511 | 1 |
| L13203 | 2,089 | | 1,131 | 5q35.1 | FOXI1_HUMAN | 169,465,551 | 169,468,951 | 1 | 2q35 | CYP27A1 | 219,387,144 | 219,387,338 | 1 |
| S46950 | 2,572 | | 1,236 | 3 | IG | 108,500,828 | 108,501,038 | 0 | 22q11.23 | ADORA2A | 23,159,092 | 23,168,300 | 1 |
| CR933675 | 6,240 | | 549 | 1 | IG | 169,858,006 | 169,859,741 | 0 | 18q21.2 | TCF4 | 51,082,825 | 51,282,368 | 1 |
| AL834489 | 3,747 | | 1,377 | 12q13.12 | FMNL3 | 48,387,256 | 48,387,569 | 1 | 5q35.2 | RNF44 | 175,886,319 | 175,891,675 | 1 |
| BX640651 | 5,566 | | 585 | 15q15.1 | INOC1 | 39,055,271 | 39,067,427 | 1 | 19q13.2 | RPS19 | 47,066,896 | 47,067,324 | 1 |
| BX640832 | 3,611 | | 297 | 9q34.2 | ABO | 135,139,403 | 135,140,444 | 1 | 11q12.3 | ASRGL1 | 61,913,180 | 61,916,635 | 1 |
| AF118063 | 2,153 | | 1,713 | 11q14.2 | PICALM | 85,377,996 | 85,378,164 | -1 | 3q22.1 | TF | 134,956,028 | 134,980,319 | 1 |
| AF119856 | 2,446 | | 1,932 | 11q13.1 | SART1 | 65,502,675 | 65,503,108 | -1 | 3p21.1 | TMEM110 | 52,822,338 | 52,834,017 | 1 |
| AF318367 | 4,109 | | 570 | 9q34.3 | TMEM141 | 138,840,870 | 138,851,272 | 1 | 16 | IG | 75,792,035 | 75,792,184 | 0 |
| BC032780 | 2,872 | | 2,568 | 16p11.2 | NP_006653.1 | 30,619,633 | 30,632,492 | 0 | 17q21.2 | CNP | 37,381,996 | 37,382,273 | 0 |
| BC048012 | 2,917 | | 2,244 | 12 | IG | 49,774,092 | 49,774,286 | 0 | 1q24.2 | KIFAP3 | 168,157,098 | 168,310,349 | 0 |
| AF026941 | 3,200 | | 1,083 | 6q23.3 | Q5SYE8_HUMAN | 138,831,964 | 138,832,566 | -1 | 2p25.2 | RSAD2 | 6,935,359 | 6,954,906 | 1 |
| AF039747 | 3,966 | | 2,364 | 12q21.2 | SYT1 | 77,797,799 | 77,798,554 | -1 | 5p14.2 | CDH10 | 24,522,967 | 24,680,618 | 1 |
| AF047715 | 2,876 | | 243 | 6q23.2 | AKAP7 | 131,613,197 | 131,646,361 | 1 | 8 | IG | 59,248,710 | 59,249,322 | 0 |
| AF055982 | 1,360 | | 807 | 5q35.3 | ZFP62_HUMAN | 180,207,918 | 180,208,403 | 1 | 19q13.41 | KLK9 | 56,191,080 | 56,196,615 | 1 |
| AF060929 | 367 | | 153 | 10p12.31 | MLLT10 | 21,863,684 | 21,900,961 | 0 | 11q14.2 | PICALM | 85,347,678 | 85,363,505 | 1 |
| AF063612 | 1,501 | | 762 | 8p22 | NP_001001927.1 | 17,656,622 | 17,656,793 | -1 | 12q24.31 | OASL | 119,942,902 | 119,961,342 | 1 |
| AF064804 | 1,875 | | 1,197 | 16q22.1 | NP_612392.1 | 69,252,652 | 69,253,127 | 0 | 6p21.1 | SUPT3H | 44,885,032 | 45,453,668 | 0 |
| AF090929 | 1,292 | | 390 | 16q23.1 | Q9NZC7-6 | 76,759,387 | 76,759,912 | -1 | 17q23.3 | CCDC44 | 59,038,378 | 59,039,457 | 0 |
| AF094517 | 2,807 | | 1,500 | 3q13.12 | IFT57 | 109,368,441 | 109,421,003 | 1 | 14q24.3 | ESRRB | 75,907,477 | 76,036,961 | 0 |
| AF115512 | 1,628 | | 669 | 7q31.1 | DNAJB9 | 107,997,616 | 108,001,380 | 1 | 1q21.2 | ENSA | 148,853,626 | 148,854,052 | 1 |
| AF124512 | 1,811 | | 1,002 | 6q21 | BVES | 105,655,145 | 105,688,065 | 1 | 7q21.3 | LMTK2 | 97,656,967 | 97,657,251 | 1 |
| AF127481 | 4,991 | | 2,997 | 15q25.3 | AKAP13 | 84,021,272 | 84,089,845 | 1 | 3q24 | C3orf58 | 145,192,529 | 145,193,171 | -1 |
| AF149297 | 2,674 | | 2,112 | 10p13 | CAMK1D | 12,912,303 | 12,912,834 | -1 | 9p13.3 | C9orf127 | 35,819,269 | 35,837,232 | 1 |
| AF153201 | 3,585 | | 1,269 | 5q13.2 | NP_620137.1 | 72,413,413 | 72,420,097 | -1 | 18q12.2 | ZNFEB_HUMAN | 31,124,276 | 31,142,390 | 1 |
| AF155110 | 4,422 | | 2,445 | Xq23 | AMMECR1 | 109,326,254 | 109,326,945 | 1 | 1q41 | KCTD3 | 213,807,508 | 213,861,696 | 1 |
| AF155114 | 2,721 | | 1,302 | 6p12.1 | FBXO9 | 53,038,206 | 53,070,643 | 0 | 17q25.3 | FASN | 77,629,503 | 77,630,540 | 1 |
| AF181071 | 3,825 | | 1,950 | 7p22.1 | EIF2AK1 | 6,029,991 | 6,065,302 | 0 | 22q12.2 | ZN278_HUMAN | 30,051,790 | 30,052,778 | 1 |
| AF209975 | 2,222 | | 363 | 8q23.1 | ANGP1_HUMAN | 108,332,784 | 108,366,165 | -1 | 17p13.3 | METT10D | 2,329,375 | 2,330,720 | 1 |
| AF216077 | 4,167 | | 498 | 9q32 | COL27A1 | 116,016,942 | 116,020,225 | 1 | 13q12.12 | TNFRSF19 | 23,147,432 | 23,148,228 | 1 |
| AF218035 | 1,721 | | 582 | 17q25.3 | ZNF750 | 78,380,631 | 78,381,365 | 0 | 7q36.1 | ABP1 | 150,185,885 | 150,189,310 | 0 |
| AF226731 | 1,114 | | 573 | 19p13.3 | M6PRBP1 | 4,789,890 | 4,790,006 | 1 | 1p13.3 | AA3R_HUMAN | 111,827,493 | 111,833,807 | 1 |
| AF230095 | 1,584 | | 855 | 11q13.4 | NUMA1 | 71,455,777 | 71,456,261 | 1 | 22q13.33 | MIOX | 49,272,166 | 49,275,346 | 1 |
| AF262027 | 2,246 | | 495 | 3q26.2 | EIF5A2 | 172,092,914 | 172,108,326 | 1 | 9q31.2 | RAD23B | 109,133,505 | 109,134,294 | 1 |
| AF286487 | 2,130 | | 1,092 | 3p21.31 | DOCK3 | 51,353,703 | 51,362,820 | -1 | 8p21.2 | PNMA2 | 26,420,715 | 26,422,444 | 1 |
| AF302785 | 1,390 | | 699 | 15q26.1 | MFGE8 | 87,247,708 | 87,248,425 | 1 | 19p13.2 | DNM2 | 10,683,835 | 10,685,040 | 1 |
| AF320070 | 3,542 | | 1,818 | 5q34 | SLIT3 | 168,130,900 | 168,132,398 | 1 | Xp11.21 | MAGED2 | 54,851,559 | 54,859,170 | 1 |
| AF321617 | 3,109 | | 534 | 1q32.1 | ELK4 | 203,843,697 | 203,845,421 | 1 | 4p15.32 | MED28 | 17,225,371 | 17,235,258 | 1 |
| AF323119 | 528 | | 138 | 20 | IG | 410,567 | 410,982 | 0 | 2q31.2 | NFE2L2 | 177,806,790 | 177,837,573 | 1 |
| AF422925 | 2,503 | | 1,728 | 2q33.1 | CASP8 | 201,831,086 | 201,859,727 | 0 | 10q23.32 | PCGF5 | 93,032,077 | 93,032,684 | 1 |
| AF466364 | 2,725 | | 414 | 8p12 | UNC5D | 35,769,883 | 35,771,716 | 1 | 17q23.3 | TEX2 | 59,578,763 | 59,579,645 | 1 |
| AF487905 | 1,874 | | 1,869 | 11q23.3 | MLL | 117,857,934 | 117,860,900 | 1 | 4q21.3 | AFF1 | 88,224,294 | 88,265,260 | 1 |
| AF488411 | 3,385 | | 264 | 8 | IG | 128,285,906 | 128,287,079 | 0 | 9p21.3 | CDKN2B | 21,992,920 | 21,995,134 | 0 |
| AF508911 | 551 | | 213 | 1 | IG | 182,564,826 | 182,568,583 | 0 | 12 | IG | 104,342,063 | 104,342,568 | 0 |
| AF533653 | 1,245 | | 546 | 12q14.3 | NP_003475.1 | 64,508,048 | 64,644,111 | 0 | 14q24.1 | RAD51L1 | 67,488,585 | 67,488,827 | 1 |
| AF542551 | 2,529 | | 1,338 | 10q26.3 | 2ABD_HUMAN | 133,604,075 | 133,619,301 | 1 | 16q13 | CPNE2 | 55,704,699 | 55,739,376 | 0 |
| AK000309 | 2,530 | | 2,208 | 6p21.31 | URFB1_HUMAN | 34,867,835 | 34,934,141 | 0 | 1p36.33 | NA | 555,640 | 555,925 | 1 |
| AK000442 | 1,573 | | 879 | 1p34.3 | RHBDL2 | 39,124,555 | 39,157,442 | 1 | 17p13.3 | NXN | 649,335 | 649,804 | 1 |
| AK000727 | 2,196 | | 423 | 11p15.1 | NP_060784.3 | 18,509,139 | 18,522,816 | 1 | 12q15 | LYZ | 68,033,811 | 68,034,280 | 1 |
| AK000903 | 1,608 | | 300 | 13q34 | COL4A1 | 109,683,116 | 109,684,525 | 1 | 3p21.1 | Q8WVI0_HUMAN | 52,545,861 | 52,549,581 | 1 |
| AK000925 | 1,599 | | 783 | 10q21.3 | RUFY2 | 69,823,527 | 69,837,040 | 1 | 2q37.3 | NP_004726.1 | 238,344,063 | 238,344,684 | 1 |
| AK001118 | 1,548 | | 234 | 7q33 | EXOC4 | 132,837,002 | 132,837,477 | 1 | 6p21.1 | UBR2_HUMAN | 42,688,817 | 42,689,893 | 1 |
| AK001125 | 2,486 | | 279 | 10q24.2 | DNMBP | 101,687,174 | 101,687,597 | 1 | 9p23 | C9orf150 | 12,806,067 | 12,808,129 | 1 |
| AK001792 | 2,081 | | 717 | 10p11.23 | SVIL | 29,853,494 | 29,879,850 | 0 | 2 | IG | 38,501,839 | 38,503,209 | 0 |
| AK021493 | 1,325 | | 228 | Xq21.1 | ZNF6 | 84,413,996 | 84,414,250 | 1 | 7p15.1 | CREB5 | 28,730,718 | 28,731,787 | 1 |
| AK021579 | 1,902 | | 213 | 12q23.1 | NP_064525.1 | 98,121,231 | 98,122,665 | 1 | 2p16.2 | ASB3 | 53,750,622 | 53,751,093 | 1 |
| AK021711 | 1,704 | | 315 | 14 | IG | 62,832,768 | 62,833,887 | 0 | 8q24.13 | ZHX1 | 124,331,281 | 124,334,866 | 1 |
| AK021880 | 1,741 | | 120 | 3q29 | CENTB2 | 196,482,564 | 196,482,852 | 1 | 6q26 | QKI | 163,851,544 | 163,853,000 | 1 |
| AK022015 | 1,772 | | 321 | 14q31.1 | SEL1L | 81,036,609 | 81,037,878 | 1 | 3 | IG | 52,717,507 | 52,718,013 | 0 |
| AK022087 | 2,431 | | 624 | 13q14.11 | DNAJC15 | 42,545,905 | 42,546,799 | 1 | 2q35 | DES | 219,993,102 | 219,999,698 | 1 |
| AK022248 | 1,801 | | 840 | 12p13.33 | TSN9_HUMAN | 3,202,581 | 3,203,077 | 1 | 14q23.1 | ACTR10 | 57,744,421 | 57,771,272 | 1 |
| AK022268 | 2,256 | | 279 | 3q26.32 | TBL1XR1 | 178,385,183 | 178,386,712 | 1 | 8q24.12 | ENPP2 | 120,712,326 | 120,713,052 | 1 |
| AK022362 | 1,797 | | 315 | 2 | IG | 58,582,448 | 58,583,919 | 0 | 6q25.3 | VIL2 | 159,139,668 | 159,140,073 | 1 |
| AK022713 | 2,199 | | 756 | 1q25.1 | DARS2 | 172,076,674 | 172,093,653 | 1 | 20p13 | CT027_HUMAN | 3,682,156 | 3,696,401 | 1 |
| AK023308 | 2,371 | | 1,851 | 1p13.1 | MAN1A2 | 117,712,088 | 117,843,551 | 1 | 3q13.2 | KIAA2018 | 114,856,965 | 114,857,924 | 1 |
| AK023578 | 2,023 | | 207 | 8q21.3 | SLC26A7 | 92,406,142 | 92,406,598 | 1 | 13q21.2 | TDRD3 | 60,044,440 | 60,046,004 | 1 |
| AK024039 | 2,224 | | 1,371 | 2p23.1 | GALNT14 | 31,000,568 | 31,214,873 | 1 | 1q22 | MAPIP_HUMAN | 154,291,211 | 154,294,920 | 1 |
| AK024312 | 1,884 | | 909 | 14q11.2 | DHRS1_HUMAN | 23,835,882 | 23,838,826 | 1 | 6p21.32 | BRD2 | 33,050,376 | 33,053,952 | 1 |
| AK024417 | 1,985 | | 345 | X | IG | 62,653,516 | 62,697,548 | 0 | 3q21.3 | RAB7 | 130,013,903 | 130,015,623 | 1 |
| AK024569 | 2,233 | | 444 | 1q42.12 | CNIH4 | 222,611,221 | 222,630,314 | 1 | 8p21.2 | PPP2R2A | 25,246,396 | 25,247,993 | 1 |
| AK024974 | 1,952 | | 516 | 15q21.3 | TCF12 | 55,027,761 | 55,028,868 | 1 | 2p11.2 | IGKC | 88,937,794 | 89,349,108 | 0 |
| AK025021 | 4,675 | | 255 | 2q32.2 | GLS | 191,505,772 | 191,508,260 | 1 | 1p13.3 | PSMA5 | 109,749,593 | 109,751,739 | 1 |
| AK025277 | 2,350 | | 366 | 4q35.1 | SORBS2 | 186,847,260 | 186,848,227 | 1 | 16p12.1 | TNRC6A | 24,736,455 | 24,737,835 | 1 |
| AK025280 | 2,656 | | 744 | 12q24.21 | THRAP2 | 114,928,995 | 114,931,333 | 1 | 1 | IG | 554,370 | 554,676 | 0 |
| AK025416 | 2,063 | | 261 | 10q23.1 | NP_997256.1 | 85,923,474 | 85,934,717 | 1 | 2 | IG | 128,668,250 | 128,669,721 | 0 |
| AK025474 | 2,378 | | 858 | 1q32.1 | RIPK5 | 203,378,248 | 203,379,037 | -1 | 17q21.2 | RAB5C | 37,530,523 | 37,536,139 | 1 |
| AK025497 | 2,503 | | 195 | Xq26.2 | PHF6 | 133,388,261 | 133,389,948 | 1 | 1p35.3 | STX12 | 28,022,075 | 28,022,867 | 1 |
| AK026001 | 1,624 | | 1,140 | 16q24.3 | ANKRD11 | 87,876,334 | 87,877,318 | 1 | 9q22.33 | HABP4 | 98,292,817 | 98,293,439 | 0 |
| AK026080 | 2,836 | | 2,628 | 11q13.1 | PCNXL3 | 65,141,035 | 65,148,580 | 0 | 22q12.2 | SF3A1 | 29,060,164 | 29,060,527 | 1 |
| AK026198 | 1,221 | | 291 | 11 | IG | 64,946,851 | 64,947,850 | 0 | 2p11.2 | IGKC | 88,937,794 | 88,937,987 | 1 |
| AK026378 | 1,332 | | 219 | 9q34.13 | CRSP8_HUMAN | 133,788,115 | 133,789,257 | 1 | 3 | IG | 109,942,884 | 109,943,055 | 0 |
| AK026383 | 2,952 | | 1,005 | 14q32.13 | DDX24 | 93,589,128 | 93,596,643 | 1 | 8q24.22 | NDRG1 | 134,318,600 | 134,329,350 | 1 |
| AK026450 | 2,217 | | 1,059 | 20q13.12 | ZSWIM1 | 43,943,280 | 43,946,351 | 1 | 8q24.3 | DGAT1 | 145,510,762 | 145,511,724 | 1 |
| AK026834 | 2,755 | | 306 | 3q28 | LPP | 190,008,606 | 190,009,826 | 1 | 1p13.1 | CD58 | 116,858,682 | 116,860,187 | 1 |
| AK027055 | 2,290 | | 555 | 9q22.2 | SPIN | 90,281,846 | 90,283,164 | 1 | 11p15.3 | USP47 | 11,928,556 | 11,934,691 | 1 |
| AK027097 | 1,363 | | 126 | 2q14.3 | MKI67IP | 122,208,822 | 122,209,893 | 1 | 22 | IG | 49,325,478 | 49,325,739 | 0 |
| AK027242 | 1,919 | | 213 | 21q21.3 | APP | 26,460,086 | 26,461,513 | 1 | 8p12 | RAB11FIP1 | 37,837,343 | 37,837,813 | 1 |
| AK027315 | 1,590 | | 495 | 2q33.1 | PPIL3 | 201,444,288 | 201,462,237 | 0 | 14q21.3 | PPIL5 | 49,135,455 | 49,151,139 | 1 |
| AK027346 | 1,459 | | 876 | 5q31.1 | PCBD2 | 134,286,910 | 134,287,078 | -1 | 6q22.33 | RSPO3 | 127,481,876 | 127,560,603 | 1 |
| AK027463 | 2,067 | | 1,479 | 17q23.3 | FTSJ3 | 59,253,179 | 59,258,188 | 1 | 1 | IG | 558,085 | 558,706 | 0 |
| AK054747 | 1,988 | | 201 | 14q23.1 | RTN1 | 59,136,907 | 59,137,561 | 1 | 8p21.2 | DPYSL2 | 26,570,268 | 26,571,607 | 1 |
| AK054867 | 1,713 | | 225 | 9q33.3 | OLFML2A | 126,613,262 | 126,614,156 | 1 | 12q24.23 | PAXI_HUMAN | 119,132,634 | 119,133,456 | 1 |
| AK054975 | 1,753 | | 411 | 6p22.1 | GABBR1 | 29,703,406 | 29,703,765 | 1 | 15q26.1 | Q6ZVC9_HUMAN | 89,641,390 | 89,642,787 | 1 |
| AK055455 | 2,185 | | 306 | 10 | IG | 76,608,074 | 76,608,377 | 0 | 14q24.1 | ZFYVE26 | 67,307,443 | 67,309,327 | 1 |
| AK056665 | 2,863 | | 1,836 | 1p21.3 | NP_055654.2 | 99,502,488 | 99,545,219 | 1 | 7 | IG | 149,701,553 | 149,702,059 | 0 |
| AK057369 | 1,819 | | 570 | Xq28 | FATE1 | 150,635,227 | 150,642,286 | 1 | 2q11.2 | TMEM131 | 97,739,235 | 97,740,087 | 1 |
| AK057440 | 1,065 | | 357 | 4 | IG | 171,190,510 | 171,190,714 | 0 | 1q42.13 | Q5T441_HUMAN | 226,418,449 | 226,419,308 | 1 |
| AK057461 | 1,844 | | 924 | 22q11.21 | C22orf25 | 18,404,441 | 18,432,882 | 1 | 20q13.13 | DPM1 | 48,997,301 | 48,997,518 | -1 |
| AK057501 | 1,743 | | 744 | 9 | IG | 6,056,135 | 6,056,829 | 0 | 16q22.1 | CMTM2 | 65,170,855 | 65,179,668 | 1 |
| AK057826 | 1,024 | | 198 | 5q35.2 | CPLX2 | 175,231,187 | 175,238,616 | 1 | 3q22.1 | RAB6B | 135,025,777 | 135,026,458 | 1 |
| AK057854 | 1,544 | | 552 | 1q21.3 | SNX27 | 149,851,113 | 149,851,571 | 1 | 5q35.2 | SNCB | 175,979,817 | 175,989,962 | 1 |
| AK057893 | 1,998 | | 747 | 9q34.3 | GPSM1 | 138,367,569 | 138,369,011 | 1 | 17q25.1 | KIAA0195 | 71,007,203 | 71,007,761 | 1 |
| AK057982 | 3,177 | | 441 | 6q21 | SNX3 | 108,640,016 | 108,689,141 | 0 | 7q31.2 | CAV1 | 115,953,813 | 115,988,466 | 1 |
| AK058132 | 1,362 | | 252 | 8q12.3 | GGH | 64,099,485 | 64,100,407 | -1 | 1q21.3 | SMCP | 151,117,421 | 151,124,145 | 1 |
| AK058161 | 967 | | 582 | 12q24.31 | CCDC62 | 121,842,517 | 121,851,853 | 1 | 11 | IG | 63,944,580 | 63,945,117 | 0 |
| AK074141 | 4,327 | | 966 | 11q13.1 | CCDC88 | 63,867,587 | 63,874,435 | 1 | 19p13.12 | CD97 | 14,377,239 | 14,379,450 | 1 |
| AK074469 | 1,919 | | 213 | 21q21.3 | APP | 26,460,086 | 26,461,513 | 1 | 8p12 | RAB11FIP1 | 37,837,343 | 37,837,813 | 1 |
| AK074489 | 2,037 | | 1,053 | 12q24.33 | NOC4L | 131,194,965 | 131,201,540 | 1 | 1q41 | RAB3GAP2 | 218,390,088 | 218,391,142 | 1 |
| AK074502 | 1,623 | | 243 | 20p11.23 | ZNF133 | 18,233,982 | 18,235,341 | 1 | 2q33.1 | BMPR2 | 203,140,459 | 203,140,715 | 1 |
| AK074580 | 2,021 | | 231 | 10q21.3 | RUFY2 | 69,822,726 | 69,823,015 | 1 | 17p13.1 | ALOX12 | 6,845,175 | 6,856,385 | -1 |
| AK075025 | 2,064 | | 351 | 14q11.2 | NM_001002000.1 | 23,771,506 | 23,777,507 | 1 | 9p13.3 | STOML2 | 35,089,893 | 35,091,490 | 1 |
| AK075051 | 2,146 | | 540 | 9q34.11 | IER5L | 130,979,929 | 130,980,361 | 0 | 14q21.3 | MGAT2 | 49,158,068 | 49,159,787 | 1 |
| AK075165 | 1,653 | | 702 | 7q31.1 | NP_031382.1 | 107,451,458 | 107,465,167 | 1 | 2q24.3 | COBLL1 | 165,249,501 | 165,250,187 | 1 |
| AK075484 | 2,308 | | 666 | 19q13.2 | SAMD4B | 44,536,484 | 44,552,595 | 0 | 2p15 | KIAA1841 | 61,196,424 | 61,245,468 | 0 |
| AK075486 | 1,706 | | 750 | 20q13.33 | ARFGAP1 | 61,390,389 | 61,391,585 | 1 | 11q13.1 | COX8A | 63,498,667 | 63,500,590 | 1 |
| AK090705 | 1,742 | | 183 | 2q34 | FYV1_HUMAN | 208,882,951 | 208,884,181 | 1 | 1 | IG | 559,102 | 559,617 | 0 |
| AK090784 | 3,049 | | 2,328 | 5 | IG | 153,456,776 | 153,509,700 | 0 | 1q42.2 | PCNXL2 | 231,186,505 | 231,256,853 | 0 |
| AK091050 | 2,391 | | 2,199 | 14q24.3 | YLPM1_HUMAN | 74,317,013 | 74,335,226 | 1 | 7q34 | EPHB6 | 142,278,684 | 142,278,967 | 1 |
| AK091155 | 3,370 | | 2,004 | 18q22.3 | TSHZ1 | 71,051,740 | 71,128,928 | 1 | Xq28 | BGN | 152,427,806 | 152,428,198 | 1 |
| AK091454 | 2,613 | | 750 | 3q27.2 | NP_001020437.1 | 186,282,555 | 186,353,496 | 1 | 19q13.33 | NUP62 | 55,101,898 | 55,102,627 | 1 |
| AK091486 | 2,486 | | 447 | 4q31.1 | CCRN4L | 140,176,044 | 140,177,023 | -1 | 10q24.1 | MMS19L | 99,208,073 | 99,210,066 | 1 |
| AK091504 | 3,218 | | 378 | 10q24.31 | SFXN3 | 102,787,139 | 102,790,381 | 1 | 7p11.2 | CHCHD2 | 56,137,177 | 56,137,643 | 1 |
| AK091514 | 2,997 | | 285 | 9q31.1 | TEX10 | 102,107,646 | 102,109,972 | 1 | 17q11.1 | WSB1 | 22,664,099 | 22,664,772 | 1 |
| AK091749 | 2,382 | | 1,659 | 6p21.2 | DAAM2 | 39,868,120 | 39,955,008 | 1 | 3q13.2 | CCDC80 | 113,806,803 | 113,811,544 | 1 |
| AK091763 | 2,432 | | 807 | 10p15.3 | DIP2C | 458,764 | 549,571 | 0 | 2 | IG | 128,112,473 | 128,127,193 | 0 |
| AK092551 | 2,353 | | 786 | 9q31.3 | CI152_HUMAN | 112,003,135 | 112,009,643 | 1 | 16p13.11 | NDE1 | 15,704,495 | 15,721,622 | 0 |
| AK092678 | 1,584 | | 906 | 11p11.2 | CKAP5 | 46,786,201 | 46,824,397 | 1 | 4q13.3 | STATH | 70,896,270 | 70,902,760 | 1 |
| AK092877 | 5,347 | | 3,375 | 19p13.2 | MYO1F | 8,492,442 | 8,548,274 | 1 | 5q33.1 | SPARC | 151,022,191 | 151,034,428 | 1 |
| AK092927 | 2,128 | | 1,074 | 7q22.1 | Q6PJS5_HUMAN | 100,515,470 | 100,518,086 | 1 | 19q13.33 | SNRP70 | 54,301,034 | 54,303,671 | 1 |
| AK092983 | 2,784 | | 1,143 | 19 | IG | 59,847,123 | 59,848,696 | 0 | 12q24.31 | UBC | 123,962,147 | 123,963,363 | 1 |
| AK093117 | 3,817 | | 1,275 | 1p36.12 | PINK1 | 20,837,002 | 20,850,397 | 0 | 3p21.31 | HYAL3 | 50,305,265 | 50,311,780 | 1 |
| AK093337 | 3,712 | | 807 | 9 | IG | 25,571,074 | 25,578,601 | 0 | 19p13.3 | BSG | 523,596 | 534,486 | 1 |
| AK093546 | 2,167 | | 363 | 22q12.3 | MYH9 | 35,060,870 | 35,062,280 | 1 | 1p36.11 | C1orf128 | 23,986,546 | 23,987,307 | 1 |
| AK093793 | 1,944 | | 630 | 17q25.3 | KIAA1618 | 75,849,260 | 75,876,352 | 1 | 9p13.3 | SIT1 | 35,639,300 | 35,640,700 | 1 |
| AK094099 | 1,899 | | 273 | 3p26.1 | ITPR1_HUMAN | 4,550,838 | 4,552,033 | 1 | 1 | IG | 558,911 | 559,617 | 0 |
| AK094100 | 1,893 | | 183 | 8q23.1 | NUDCD1 | 110,378,123 | 110,379,504 | 1 | 1 | IG | 559,100 | 559,615 | 0 |
| AK094274 | 2,622 | | 1,182 | 17p11.2 | NP_001028725.1 | 19,853,206 | 20,076,310 | 0 | 18q23 | PQLC1 | 75,763,409 | 75,763,761 | 1 |
| AK094335 | 1,882 | | 369 | 13q14.3 | VPS36 | 51,902,086 | 51,903,269 | 1 | 2p13.3 | Q9ULR5_HUMAN | 71,268,946 | 71,307,709 | 1 |
| AK094455 | 1,561 | | 534 | 11p15.5 | Q6ZTG5_HUMAN | 849,079 | 849,787 | 0 | 14q32.2 | SLC25A29 | 99,832,707 | 99,833,711 | 1 |
| AK094473 | 2,300 | | 378 | 4q21.1 | Sep-11 | 78,155,095 | 78,161,816 | 1 | 1q22 | SYT11 | 154,120,648 | 154,121,606 | 1 |
| AK094526 | 2,214 | | 723 | 4q21.23 | WDFY3 | 85,914,997 | 85,923,203 | 1 | 21 | IG | 21,037,114 | 21,097,235 | 0 |
| AK094555 | 4,688 | | 1,827 | 10q24.32 | PSD | 104,162,145 | 104,171,286 | 0 | 14q32.13 | DDX24 | 93,587,021 | 93,615,666 | -1 |
| AK094603 | 2,650 | | 1,050 | 1q32.1 | IPO9 | 200,064,958 | 200,090,590 | 1 | 4q35.1 | SORBS2 | 186,743,672 | 186,745,484 | 1 |
| AK095454 | 1,974 | | 900 | 14q24.3 | LTBP2 | 74,108,214 | 74,108,549 | 1 | 15q15.1 | PLCB2 | 38,367,401 | 38,370,822 | 1 |
| AK095520 | 2,459 | | 285 | 15q24.3 | TSPAN3 | 75,125,716 | 75,150,506 | 1 | 14q31.3 | FLRT2 | 85,162,903 | 85,164,011 | 1 |
| AK095613 | 2,332 | | 324 | 20q11.21 | CT112_HUMAN | 30,536,758 | 30,538,507 | -1 | 9 | IG | 124,619,611 | 124,620,195 | 0 |
| AK095618 | 2,710 | | 351 | 16q22.2 | XP_946970.1 | 71,020,753 | 71,125,289 | -1 | 9q34.11 | TOR1B | 131,606,988 | 131,608,057 | 1 |
| AK095664 | 4,027 | | 432 | 1 | IG | 23,115,251 | 23,119,934 | 0 | 7q11.23 | TMEM60 | 77,260,982 | 77,261,414 | 1 |
| AK096494 | 2,818 | | 828 | 20p11.21 | NAPB | 23,308,096 | 23,350,103 | 0 | 17q12 | ZNHIT3 | 31,916,635 | 31,929,267 | 0 |
| AK096568 | 2,858 | | 792 | 5q35.2 | ARL10 | 175,725,111 | 175,733,109 | 1 | 20 | IG | 36,848,040 | 36,848,502 | 0 |
| AK096600 | 2,827 | | 1,830 | 19p13.3 | DIRAS1 | 2,668,155 | 2,672,361 | 1 | 1q21.3 | CGN | 149,766,075 | 149,776,590 | 0 |
| AK097113 | 2,187 | | 750 | 20p13 | Q5JXA9_HUMAN | 1,418,878 | 1,420,169 | 1 | 6p21.33 | HSPA1A | 31,892,796 | 31,893,696 | 1 |
| AK097596 | 1,853 | | 642 | 20q11.23 | Q7Z4R2_HUMAN | 36,508,732 | 36,509,555 | 1 | 8q24.3 | PARP10 | 145,123,332 | 145,131,231 | 1 |
| AK097626 | 2,462 | | 288 | 11p11.2 | ACP2 | 47,225,014 | 47,226,932 | 1 | 8p11.21 | INDOL1 | 39,880,451 | 39,971,973 | 0 |
| AK097630 | 1,963 | | 834 | 17q23.2 | HSF5 | 53,854,147 | 53,899,310 | 1 | 11q23.3 | TRAPPC4 | 118,396,087 | 118,399,585 | 1 |
| AK097726 | 1,785 | | 633 | 10 | IG | 91,449,261 | 91,451,143 | 0 | 2p24.3 | NP_056993.2 | 15,224,489 | 15,296,269 | 0 |
| AK097768 | 1,532 | | 921 | Xp11.4 | BCOR | 39,806,541 | 39,807,142 | 1 | 7p12.1 | NP_872401.2 | 53,070,829 | 53,072,111 | 1 |
| AK098118 | 2,662 | | 348 | 17q24.2 | PRKCA | 62,113,099 | 62,115,135 | 1 | 12q15 | LYZ | 68,033,650 | 68,034,280 | 1 |
| AK098133 | 2,322 | | 756 | 1p13.3 | U6 | 111,994,656 | 111,995,173 | 0 | 3q27.1 | EIF4G1 | 185,528,599 | 185,535,536 | 1 |
| AK098190 | 2,199 | | 1,254 | 5q31.1 | KLHL3 | 137,000,882 | 137,099,603 | 1 | 15 | IG | 39,906,709 | 39,907,343 | 0 |
| AK098204 | 3,190 | | 303 | 11q21 | C11orf75 | 92,852,644 | 92,854,433 | 1 | 7q11.23 | BCL7B | 72,588,622 | 72,592,211 | 1 |
| AK122613 | 2,981 | | 2,145 | 3q29 | ATP13A5 | 194,485,576 | 194,579,326 | 1 | 1 | IG | 558,891 | 559,617 | 0 |
| AK122627 | 5,671 | | 1,257 | 5q32 | GPR151 | 145,872,859 | 145,875,946 | 1 | 16p11.2 | C16orf58 | 31,408,316 | 31,426,968 | 0 |
| AK122725 | 2,395 | | 1,596 | 22q11.21 | MICAL3 | 16,754,352 | 16,887,325 | 0 | 16p13.3 | HN1L_HUMAN | 1,690,975 | 1,691,428 | 1 |
| AK122822 | 3,674 | | 2,133 | 6q12 | BAI3 | 69,638,451 | 69,638,920 | 1 | 17p13.1 | ALOXE3 | 7,939,944 | 7,962,556 | 1 |
| AK123035 | 1,778 | | 489 | 21q22.12 | RUNX1 | 35,181,213 | 36,278,878 | 0 | 17q21.2 | CCR10 | 38,085,493 | 38,085,946 | -1 |
| AK123065 | 1,802 | | 717 | 4 | IG | 106,228,040 | 106,228,838 | 0 | 17p13.2 | SPAG7 | 4,803,246 | 4,811,832 | 1 |
| AK123135 | 1,968 | | 1,656 | 6q25.1 | SYNE1 | 152,754,158 | 152,772,462 | 1 | 8q24.3 | KIFC2 | 145,669,979 | 145,670,306 | 1 |
| AK123145 | 1,734 | | 378 | 1p36.22 | Q6ZWG2_HUMAN | 9,164,652 | 9,185,265 | 1 | 10 | IG | 53,736,198 | 53,739,310 | 0 |
| AK123498 | 2,440 | | 675 | 2p16.3 | LHCGR | 48,712,934 | 48,836,367 | 0 | 6p22.2 | C6orf62 | 24,813,071 | 24,813,406 | 1 |
| AK123514 | 1,937 | | 369 | 8q13.1 | NP_062553.1 | 67,742,349 | 67,743,219 | 1 | 5q13.2 | RAD17 | 68,702,141 | 68,705,670 | 1 |
| AK123590 | 2,894 | | 1,311 | Xp11.22 | HUWE1_HUMAN | 53,689,095 | 53,727,806 | 1 | 20q13.12 | PIGT | 43,478,195 | 43,488,297 | 0 |
| AK123678 | 3,081 | | 735 | 12q13.2 | ITGA5 | 53,075,312 | 53,076,328 | -1 | 3p24.1 | NP_071906.1 | 28,339,867 | 28,365,290 | 0 |
| AK124078 | 1,500 | | 216 | 22q13.2 | XRCC6 | 40,386,391 | 40,386,843 | 1 | 14q32.33 | SIVA_HUMAN | 104,295,985 | 104,297,035 | 1 |
| AK124325 | 1,766 | | 417 | 4q33 | Q6ZVM8_HUMAN | 171,185,909 | 171,186,538 | 1 | 1q43 | MTR | 235,129,145 | 235,130,285 | 1 |
| AK124345 | 2,368 | | 1,350 | 1p34.3 | MACF1 | 39,650,503 | 39,651,830 | 1 | 11 | IG | 64,949,048 | 64,950,572 | 0 |
| AK124719 | 3,119 | | 507 | 12 | IG | 127,059,708 | 127,060,312 | 0 | 17p13.2 | Q8TE90_HUMAN | 3,854,501 | 3,857,020 | 0 |
| AK125076 | 3,676 | | 2,034 | 18q21.32 | CCBE1 | 55,495,019 | 55,495,763 | 1 | 7q22.1 | CUTL1 | 101,246,005 | 101,713,969 | 0 |
| AK125291 | 1,974 | | 174 | 5 | IG | 139,955,567 | 139,956,660 | 0 | 7q32.1 | NP_789784.2 | 126,797,652 | 126,800,769 | 1 |
| AK125614 | 2,191 | | 408 | 9q22.2 | SECISBP2 | 91,161,261 | 91,162,857 | 1 | Xp11.23 | RBM3 | 48,319,722 | 48,321,615 | 1 |
| AK126049 | 3,538 | | 711 | 10q26.3 | MGMT | 131,208,203 | 131,210,381 | 1 | 17q21.2 | ACLY | 37,276,698 | 37,281,579 | 1 |
| AK126071 | 3,515 | | 819 | 15q24.1 | NP_001035826.1 | 71,522,558 | 71,639,396 | 1 | 4p14 | NP_612398.1 | 38,609,567 | 38,623,755 | 1 |
| AK126172 | 2,253 | | 426 | 22q12.2 | ASCC2 | 28,562,680 | 28,564,142 | 1 | 6p21.1 | FOXP4 | 41,677,303 | 41,678,099 | 1 |
| AK126272 | 2,612 | | 1,785 | 3q26.31 | NAALADL2 | 176,064,310 | 176,781,703 | 0 | 12q15 | LYZ | 68,033,757 | 68,034,280 | 1 |
| AK126476 | 4,002 | | 1,062 | 19p13.11 | COPE | 18,871,391 | 18,891,184 | 0 | 3p21.31 | PLXNB1 | 48,420,266 | 48,423,375 | 1 |
| AK126960 | 2,737 | | 378 | 15q21.3 | Q6ZT32_HUMAN | 52,173,379 | 52,175,426 | 1 | 1q25.3 | NMNAT2 | 181,483,996 | 181,484,692 | 1 |
| AK127078 | 3,814 | | 2,532 | 15q13.3 | Q6ZSY1_HUMAN | 30,853,029 | 31,146,321 | 0 | 12q13.3 | ATP5B | 55,318,229 | 55,320,090 | 1 |
| AK127081 | 4,973 | | 2,148 | 10q22.2 | USP54 | 74,928,443 | 74,971,466 | 0 | 7 | IG | 47,961,486 | 47,962,402 | 0 |
| AK127106 | 3,279 | | 2,871 | 19p13.11 | UNC13A | 17,589,616 | 17,620,417 | 1 | 17q25.1 | WBP2 | 71,353,420 | 71,353,889 | 1 |
| AK127175 | 2,814 | | 606 | 17q25.1 | DNAI2 | 69,804,128 | 69,804,872 | 1 | 1p32.3 | ECHDC2 | 53,134,493 | 53,136,567 | 1 |
| AK127230 | 4,505 | | 324 | 15 | IG | 70,866,529 | 70,869,641 | 0 | 11p15.3 | EIF4G2 | 10,775,178 | 10,776,577 | 1 |
| AK127347 | 3,932 | | 483 | 19 | IG | 55,683,511 | 55,684,398 | 0 | 10p15.3 | Q6ZSL0_HUMAN | 1,213,542 | 1,216,584 | 1 |
| AK128498 | 3,297 | | 1,473 | 8q24.11 | EXT1 | 118,990,033 | 118,990,884 | 1 | 18p11.32 | CLUL1 | 587,674 | 635,227 | 1 |
| AK128501 | 3,481 | | 720 | 22q11.23 | BCR | 21,955,396 | 21,963,318 | 1 | 3 | IG | 198,822,801 | 198,823,415 | 0 |
| AK128886 | 2,263 | | 342 | 8 | IG | 16,413,226 | 16,469,482 | 0 | 5q31.1 | PCBD2 | 134,290,248 | 134,291,696 | -1 |
| AK129536 | 1,590 | | 219 | 2q22.3 | ZFHX1B | 144,983,706 | 144,984,056 | 1 | 1p36.11 | PAFAH2 | 26,158,847 | 26,160,091 | 1 |
| AK129562 | 1,810 | | 213 | 20p12.3 | CRLS1 | 5,956,431 | 5,957,972 | 1 | 15q21.3 | MNS1 | 54,508,299 | 54,508,564 | 0 |
| AK129614 | 1,824 | | 234 | 5q23.2 | CSNK1G3 | 122,964,334 | 122,965,636 | 1 | 4q13.3 | HTN3 | 70,928,765 | 70,936,836 | 1 |
| AK129748 | 2,820 | | 993 | 9q34.3 | BTBD14A | 138,038,204 | 138,126,952 | 1 | 1p36.11 | STMN1 | 26,099,646 | 26,105,497 | 1 |
| AK129788 | 1,110 | | 516 | 9 | IG | 10,997,906 | 10,998,771 | 0 | 7q11.23 | ELN | 73,121,923 | 73,122,170 | 1 |
| AK129856 | 1,696 | | 987 | 2q31.2 | TTN | 179,145,679 | 179,146,615 | 1 | 16q21 | GOT2 | 57,298,538 | 57,299,301 | 1 |
| AK129876 | 1,828 | | 1,728 | 2q31.2 | TTN | 179,139,743 | 179,140,311 | 1 | 19q13.33 | HRC | 54,346,270 | 54,349,404 | 1 |
| AK129920 | 2,588 | | 504 | 12q14.2 | RASSF3 | 63,362,870 | 63,375,376 | 1 | 14q21.3 | SOS2 | 49,653,603 | 49,655,124 | 1 |
| AK129954 | 1,352 | | 252 | 3p14.2 | PTPRG | 61,758,357 | 61,759,240 | 1 | 1 | IG | 555,456 | 555,922 | 0 |
| AK130007 | 1,856 | | 1,722 | 17q11.2 | KIAA0100 | 23,989,167 | 23,996,259 | 1 | 1p34.3 | TRAPPC3 | 36,375,224 | 36,375,439 | 1 |
| AK130038 | 1,933 | | 210 | 10 | IG | 69,700,899 | 69,701,701 | 0 | 5p13.2 | UGT3A1 | 35,988,967 | 35,990,100 | 1 |
| AK130086 | 1,696 | | 198 | 5q31.1 | C5orf24 | 134,220,702 | 134,221,926 | 1 | 12q13.12 | LIMA1 | 48,902,227 | 48,902,697 | 1 |
| AK130141 | 1,447 | | 975 | 1p36.23 | UTS2 | 7,827,651 | 7,827,824 | 0 | 11q12.2 | SLC15A3 | 60,461,161 | 60,470,824 | 0 |
| AK130181 | 2,027 | | 612 | 11p11.2 | SLC35C1 | 45,783,276 | 45,784,463 | 1 | 2 | IG | 192,266,167 | 192,269,630 | 0 |
| AK130182 | 1,568 | | 1,440 | 15q21.2 | DMXL2 | 49,616,109 | 49,625,119 | 1 | 1q32.1 | LGTN | 204,831,605 | 204,837,168 | 1 |
| AK130291 | 4,590 | | 348 | 15q26.2 | MCTP2 | 92,653,481 | 92,657,831 | 1 | 11q23.1 | CRYAB | 111,285,343 | 111,286,872 | -1 |
| AK130342 | 3,285 | | 735 | 1p22.1 | CCDC18 | 93,485,063 | 93,516,755 | 1 | 7 | IG | 65,969,355 | 65,974,207 | 0 |
| AK130344 | 2,251 | | 417 | 2q32.3 | TMEFF2 | 192,622,336 | 192,623,329 | 1 | 7p14.3 | PTHB1_HUMAN | 33,375,177 | 33,376,433 | 1 |
| AK130402 | 1,340 | | 231 | 8q24.11 | EXT1 | 119,011,488 | 119,011,890 | 1 | 14q13.2 | CN024_HUMAN | 34,621,408 | 34,622,351 | 1 |
| AK130487 | 1,653 | | 747 | 7q34 | PIP | 142,539,292 | 142,546,956 | 1 | 20q11.21 | C20orf70 | 31,219,626 | 31,232,878 | 1 |
| AK130503 | 2,499 | | 216 | 15q21.3 | CGNL1 | 55,601,703 | 55,603,677 | 1 | 4q13.3 | HTN3 | 70,928,765 | 70,936,836 | 1 |
| AK130814 | 1,987 | | 1,791 | 9q31.1 | ABCA1 | 106,616,554 | 106,627,989 | 1 | Xq12 | LAS1L | 64,649,188 | 64,654,675 | 1 |
| AK130929 | 1,761 | | 240 | 1 | IG | 71,018,729 | 71,020,169 | 0 | 14q32.32 | MARK3 | 103,039,590 | 103,039,917 | 1 |
| AK130943 | 2,746 | | 591 | 1p32.3 | MAGOH | 53,473,453 | 53,474,743 | 0 | 19p13.3 | SH3GL1 | 4,334,396 | 4,335,856 | 1 |
| AK131288 | 2,136 | | 426 | 11q13.4 | Q6ZNB5_HUMAN | 71,305,289 | 71,317,136 | 1 | 1 | IG | 559,043 | 559,617 | 0 |
| AK131570 | 1,670 | | 381 | 8 | IG | 101,780,363 | 101,781,343 | 0 | 17 | IG | 37,807,995 | 37,808,691 | 0 |
| AK172771 | 1,740 | | 507 | 13q12.2 | LNX2 | 27,019,978 | 27,025,559 | 1 | 5q35.1 | BNIP1 | 172,509,833 | 172,510,492 | 1 |
| AK172796 | 2,020 | | 405 | 2q31.1 | NP_085153.1 | 176,500,516 | 176,500,811 | 1 | 1p35.3 | NP_001003682.1 | 29,317,424 | 29,319,137 | 0 |
| AK222631 | 1,979 | | 1,611 | 5p15.2 | ANKH | 14,764,324 | 14,924,585 | 0 | 15q24.3 | TSPAN3 | 75,125,425 | 75,125,898 | 1 |
| AK223175 | 1,284 | | 1,065 | 7q21.3 | COL1A2 | 93,862,143 | 93,876,072 | 1 | 10q25.3 | PNLIP | 118,309,989 | 118,317,360 | 1 |
| AK223377 | 2,035 | | 1,401 | 5q33.3 | Q96PV3_HUMAN | 159,457,647 | 159,457,770 | 1 | 11p15.4 | CK016_HUMAN | 8,898,199 | 8,911,101 | 0 |
| AB001915 | 1,633 | | 855 | 11q13.1 | NR_002819.1 | 65,024,364 | 65,024,561 | 1 | 1p22.3 | DDAH1 | 85,559,268 | 85,703,411 | 0 |
| AB011119 | 5,658 | | 2,058 | 15q15.3 | LCMT2 | 41,405,542 | 41,410,020 | 1 | 4 | IG | 68,321,423 | 68,322,601 | 0 |
| AB011149 | 5,134 | | 3,123 | 4q13.2 | YTHDC1 | 68,861,656 | 68,878,548 | -1 | 6p21.33 | DHX16 | 30,728,884 | 30,748,736 | 0 |
| AB019489 | 2,399 | | 1,131 | 8q21.11 | ZFHX4 | 77,851,372 | 77,851,886 | -1 | 1q25.1 | RABGAP1L | 172,936,781 | 173,226,347 | 1 |
| AB019524 | 6,541 | | 4,401 | 17p11.2 | NCOR1 | 15,875,622 | 15,935,998 | 1 | 1q42.13 | Q5TA31_HUMAN | 226,743,284 | 226,749,292 | 1 |
| AB024690 | 3,374 | | 1,851 | 2q31.1 | TLK1 | 171,658,547 | 171,658,815 | 1 | 11q13.1 | SYVN1 | 64,651,327 | 64,658,544 | 0 |
| AB033098 | 3,902 | | 3,069 | 20p11.23 | NP_065076.1 | 20,401,475 | 20,513,599 | 1 | 8 | IG | 85,321,489 | 85,322,323 | 0 |
| AB045369 | 2,050 | | 1,359 | 11q24.2 | NRGN | 124,121,773 | 124,121,937 | -1 | 20q13.33 | HRH3 | 60,223,412 | 60,228,449 | 1 |
| AB073653 | 3,150 | | 456 | 10q22.1 | C10orf54 | 73,188,381 | 73,192,273 | 0 | 12q24.31 | ZNF664 | 123,064,731 | 123,065,926 | 1 |
| AB073889 | 2,091 | | 219 | 8p12 | NRG1 | 32,279,121 | 32,279,982 | 1 | 10 | IG | 104,513,803 | 104,515,005 | 0 |
| AB208969 | 6,599 | | 2,940 | 1q44 | TRIM58 | 246,087,130 | 246,094,678 | 1 | 16q24.1 | KIAA0182 | 84,248,383 | 84,267,311 | 0 |
| AB209076 | 4,512 | | 681 | 2q11.1 | KCNIP3 | 95,326,813 | 95,343,371 | 1 | 11q13.4 | PDE2A | 72,038,732 | 72,039,591 | 1 |
| AB209289 | 3,321 | | 1,026 | 3q13.32 | IGSF11 | 120,173,639 | 120,174,271 | -1 | 11q13.1 | PPP2R5B | 64,448,774 | 64,458,521 | 0 |
| AB209679 | 8,198 | | 1,203 | 11q23.1 | DIXDC1 | 111,314,361 | 111,322,436 | 1 | Xq27.1 | MCF2 | 138,491,596 | 138,516,067 | 1 |
| AB209699 | 7,533 | | 525 | 12q14.1 | TSPAN31 | 56,425,108 | 56,428,219 | 1 | Xq23 | PAK3 | 110,263,678 | 110,269,215 | -1 |
| AB209779 | 2,222 | | 393 | 6p25.2 | NQO2 | 2,933,222 | 2,960,939 | 0 | 4q25 | ANK2 | 114,286,296 | 114,287,369 | 1 |
| AY004251 | 792 | | 582 | 21q22.12 | RUNX1 | 35,153,640 | 35,181,279 | 1 | 19q13.43 | NALP4 | 61,084,442 | 61,089,101 | -1 |
| AY029161 | 1,878 | | 1,065 | 8p23.1 | PINX1_HUMAN | 10,660,095 | 10,734,741 | 1 | 9q34.3 | PHPT1 | 138,863,719 | 138,865,297 | 1 |
| AY093608 | 438 | | 180 | 10 | IG | 15,285,503 | 15,285,727 | 0 | 16p12.2 | NP_057109.2 | 21,518,580 | 21,531,592 | 1 |
| AY138860 | 379 | | 375 | 12q14.3 | NP_003475.1 | 64,518,571 | 64,643,421 | 0 | 14q24.1 | RAD51L1 | 67,828,354 | 67,947,969 | 0 |
| AY189286 | 3,260 | | 561 | 3p21.1 | NP_660305.2 | 52,299,740 | 52,302,531 | 1 | 1 | IG | 26,424,667 | 26,428,534 | 0 |
| AY227114 | 948 | | 768 | 20q13.12 | STK4 | 43,057,199 | 43,063,410 | 1 | 5 | IG | 150,726,060 | 150,726,412 | 0 |
| AY305873 | 1,682 | | 879 | 20q13.32 | RAB22A | 56,369,019 | 56,369,601 | 1 | 16p13.3 | MPG | 68,245 | 75,845 | 0 |
| AY358205 | 1,233 | | 258 | 19p13.3 | MLLT1 | 6,214,092 | 6,214,480 | 1 | 8p12 | SPFH2 | 37,732,749 | 37,733,597 | 1 |
| AY358467 | 3,580 | | 1,044 | 15q15.2 | CDAN1_HUMAN | 40,803,052 | 40,810,255 | 1 | 14q32.12 | RIN3 | 92,224,148 | 92,225,089 | -1 |
| AY358504 | 2,456 | | 1,635 | 13q12.13 | NUPL1 | 24,773,774 | 24,780,089 | 1 | 16q22.1 | NP_776176.2 | 65,587,029 | 65,601,139 | 1 |
| AY358856 | 2,473 | | 2,184 | 12p13.31 | CD163L1 | 7,423,061 | 7,487,994 | 1 | 5q31.1 | PCBD2 | 134,290,250 | 134,290,566 | -1 |
| AY387666 | 941 | | 210 | 12q14.3 | Q6ZQU9_HUMAN | 64,546,850 | 64,561,625 | 0 | 14q24.1 | RAD51L1 | 68,019,048 | 68,019,200 | -1 |
| AY491779 | 3,715 | | 891 | 8p21.2 | ADRA1A | 26,777,099 | 26,778,839 | 1 | 20q11.21 | BCL2L1 | 29,769,631 | 29,771,548 | 1 |
| AY789120 | 663 | | 282 | 22q11.23 | BCR | 21,961,731 | 21,961,881 | 1 | 9q34.12 | ABL1 | 132,718,911 | 132,719,423 | 1 |
| BC001198 | 2,317 | | 1,374 | 20q11.22 | PHF20 | 33,922,416 | 33,964,675 | 0 | 19q13.2 | NP_060505.1 | 46,629,063 | 46,636,153 | 0 |
| BC001974 | 2,445 | | 1,494 | 8q24.3 | ZC3H3 | 144,590,972 | 144,689,768 | 1 | 2q11.2 | TXNDC9 | 99,301,973 | 99,302,468 | 1 |
| BC004934 | 2,227 | | 1,551 | 19p13.3 | PRSS15 | 5,642,845 | 5,656,823 | 1 | 2p13.1 | MTHFD2 | 74,295,531 | 74,295,920 | 1 |
| BC007006 | 996 | | 150 | 17q24.2 | KPNA2 | 63,462,310 | 63,463,954 | 1 | Xq22.2 | WBP5 | 102,499,300 | 102,500,044 | 1 |
| BC007395 | 2,527 | | 822 | 9q22.33 | TDRD7 | 99,285,021 | 99,298,228 | 1 | 2p23.2 | PLB1 | 28,671,020 | 28,672,434 | 1 |
| BC007937 | 3,399 | | 1,419 | 19p13.2 | PPAN | 10,078,026 | 10,082,970 | 0 | 16q12.2 | SLC6A2 | 54,296,119 | 54,297,835 | 1 |
| BC014227 | 2,667 | | 1,767 | 6q23.3 | KIAA1244 | 138,686,499 | 138,699,715 | 1 | 19q13.43 | HKR2_HUMAN | 63,517,962 | 63,530,094 | -1 |
| BC017792 | 974 | | 249 | 4p15.33 | Q9H8A7_HUMAN | 15,089,938 | 15,091,946 | 1 | 11 | IG | 22,601,184 | 22,601,868 | 0 |
| BC019032 | 2,391 | | 618 | 6p12.2 | TRAM2 | 52,473,277 | 52,474,451 | -1 | Xq23 | NP_115603.2 | 109,306,359 | 109,307,551 | 1 |
| BC024302 | 4,707 | | 1,017 | 3p21.31 | MAP4 | 47,867,189 | 47,892,315 | 1 | 2 | IG | 44,400,292 | 44,401,578 | 0 |
| BC028143 | 1,410 | | 705 | 4q28.2 | PGRMC2 | 129,428,187 | 129,428,403 | 1 | 1q42.3 | NA | 232,809,286 | 232,811,077 | 0 |
| BC033191 | 2,667 | | 1,767 | 6q23.3 | KIAA1244 | 138,686,499 | 138,699,715 | 1 | 19q13.43 | HKR2_HUMAN | 63,517,962 | 63,530,094 | -1 |
| BC035653 | 1,650 | | 447 | 17p13.2 | TAX1BP3 | 3,517,377 | 3,518,603 | 1 | 21q22.3 | UBE2G2 | 45,013,390 | 45,013,772 | 1 |
| BC036413 | 2,863 | | 246 | 12 | IG | 50,187,774 | 50,190,274 | 0 | 6q13 | LMBRD1 | 70,442,470 | 70,442,813 | 1 |
| BC037414 | 2,357 | | 561 | 7q33 | CREB3L2 | 137,215,756 | 137,236,673 | 1 | 6p21.31 | CDKN1A | 36,760,107 | 36,763,087 | 1 |
| BC038772 | 1,678 | | 513 | 18q12.2 | KIAA1328 | 32,663,076 | 32,719,797 | 0 | 8q11.21 | MCM4 | 49,052,261 | 49,053,652 | 1 |
| BC040538 | 3,824 | | 291 | 18p11.21 | CR001_HUMAN | 13,317,065 | 13,319,979 | 1 | 14q13.2 | CN024_HUMAN | 34,621,456 | 34,622,335 | 1 |
| BC040590 | 3,038 | | 381 | Xp11.3 | UBE1 | 46,935,766 | 46,937,008 | 1 | 17p13.3 | NP_055668.1 | 2,227,425 | 2,231,098 | 1 |
| BC041619 | 3,199 | | 1,437 | 11q13.1 | NP_055919.1 | 64,435,209 | 64,441,238 | 1 | 12q13.11 | FAM113B | 45,896,634 | 45,916,710 | 1 |
| BC045531 | 2,118 | | 678 | 9q34.3 | TRAF2 | 138,912,985 | 138,913,946 | 1 | 10q26.3 | 2ABD_HUMAN | 133,597,950 | 133,620,044 | 1 |
| BC047623 | 2,858 | | 246 | 12 | IG | 50,187,774 | 50,190,274 | 0 | 6q13 | LMBRD1 | 70,442,470 | 70,442,813 | 1 |
| BC047770 | 1,902 | | 654 | 1p36.22 | H6PD | 9,217,520 | 9,218,308 | 1 | 17p13.3 | NP_001020630.1 | 2,266,093 | 2,267,194 | 1 |
| BC053934 | 1,939 | | 669 | 2p14 | NP_001004345.1 | 65,522,950 | 65,537,150 | -1 | 12q24.31 | SIRT4 | 119,214,576 | 119,235,424 | 0 |
| BC057227 | 1,059 | | 951 | 1q23.3 | F11R | 159,257,423 | 159,257,592 | 1 | 3q13.33 | GOLGB1 | 122,895,379 | 122,896,238 | 1 |
| BC064616 | 2,473 | | 1,305 | 3p23 | DYNC1LI1 | 32,562,349 | 32,587,313 | 1 | 2q21.3 | ZRANB3 | 135,673,328 | 135,704,678 | 1 |
| BC066892 | 2,011 | | 261 | 16q23.1 | Q9NZC7-6 | 76,758,115 | 76,759,657 | -1 | 13 | IG | 67,051,357 | 67,055,364 | 0 |
| BC068076 | 1,450 | | 267 | 7q32.1 | Q8NBE0_HUMAN | 128,294,963 | 128,296,024 | 1 | 16q22.1 | CKLF | 65,153,568 | 65,153,949 | 1 |
| BC070300 | 761 | | 294 | 16q22.2 | HP | 70,646,026 | 70,647,958 | 1 | 6q25.3 | SLC22A3 | 160,792,857 | 160,793,447 | 1 |
| BC072022 | 1,550 | | 951 | 5p13.1 | NP_787117.3 | 41,954,500 | 41,954,843 | 1 | 19q13.31 | APOE | 50,101,688 | 50,104,490 | 1 |
| BC073739 | 647 | | 291 | 7q21.3 | PON2 | 94,883,487 | 94,902,233 | 1 | 16q22.1 | Q9H9K1_HUMAN | 67,561,139 | 67,561,534 | -1 |
| BC073825 | 2,641 | | 966 | 7q34 | ZYX | 142,790,045 | 142,798,326 | -1 | 19p13.3 | SH3GL1 | 4,311,367 | 4,312,374 | 1 |
| BC073955 | 1,660 | | 261 | Xq13.1 | OGT1_HUMAN | 70,669,696 | 70,669,895 | 1 | 9 | IG | 33,242,469 | 33,243,886 | 0 |
| BC078139 | 2,319 | | 648 | 8q24.3 | EIF2C2 | 141,677,258 | 141,679,024 | -1 | 15 | IG | 53,282,456 | 53,282,975 | 0 |
| BC080549 | 2,270 | | 1,278 | 14q32.33 | IGHG1 | 105,391,370 | 105,392,836 | -1 | 19p13.3 | C19orf6 | 960,650 | 962,095 | 0 |
| BC080565 | 3,615 | | 1,647 | 2q23.3 | NP_443137.2 | 152,900,268 | 153,183,703 | 0 | 5p15.2 | DAP | 10,732,342 | 10,801,350 | 1 |
| BC082977 | 2,046 | | 687 | 16q13 | Q86VG7_HUMAN | 56,402,050 | 56,408,351 | 1 | 11p15.5 | EPS8L2 | 717,063 | 717,724 | 1 |
| BC088728 | 3,055 | | 492 | 19 | IG | 39,686,937 | 39,689,098 | 0 | 6p24.3 | MUTED | 7,826,749 | 7,827,626 | 0 |
| BC093087 | 1,904 | | 501 | 20p13 | SLC23A2 | 4,899,437 | 4,930,125 | 1 | 9 | IG | 89,024,608 | 89,070,230 | 0 |
| BC094690 | 1,658 | | 1,119 | 19q13.33 | MYBPC2 | 55,627,972 | 55,637,347 | 1 | 9q34.3 | PTGDS | 138,991,883 | 138,996,015 | 1 |
| BC094870 | 2,579 | | 330 | 7 | IG | 156,504,283 | 156,505,952 | 0 | 4 | IG | 177,489,795 | 177,490,697 | 0 |
| BC105970 | 1,278 | | 687 | 7q22.1 | COPS6 | 99,524,578 | 99,525,305 | 1 | 19q13.2 | TIM50_HUMAN | 44,664,477 | 44,672,402 | 1 |
| BC108670 | 1,537 | | 1,131 | 12q23.1 | NP_001006948.1 | 99,060,500 | 99,060,757 | 1 | 3p14.3 | PXK | 58,326,639 | 58,370,548 | 1 |
| BC111480 | 3,211 | | 2,238 | 8q22.1 | INTS8 | 95,904,710 | 95,941,696 | 1 | 17 | IG | 53,435,856 | 53,436,155 | 0 |
| BT006944 | 1,860 | | 1,857 | 8p21.2 | DPYSL2 | 26,491,688 | 26,561,140 | 1 | 22q11.21 | SDF2L1 | 20,326,618 | 20,328,462 | 1 |
| D50682 | 1,225 | | 306 | 12 | IG | 24,224,154 | 24,225,130 | 0 | 3p24.1 | TGFBR2 | 30,666,831 | 30,688,255 | 1 |
| D50683 | 5,759 | | 1,701 | 2p22.2 | STRN | 36,949,519 | 36,951,040 | 1 | 3p24.1 | TGFBR2 | 30,623,325 | 30,710,579 | 1 |
| DQ104207 | 2,331 | | 2,328 | 8p11.21 | HOOK3 | 42,871,432 | 42,942,515 | 0 | 10q11.21 | RET | 42,932,036 | 42,943,723 | 1 |
| DQ299936 | 409 | | 405 | 4p12 | Q6AI58_HUMAN | 48,227,930 | 48,228,119 | 1 | 11q23.3 | MLL | 117,864,537 | 117,865,788 | 1 |
| DQ299938 | 492 | | 489 | 11q23.3 | MLL | 117,860,137 | 117,860,900 | 1 | 4p12 | Q6AI58_HUMAN | 48,224,390 | 48,225,112 | 1 |
| DQ299939 | 378 | | 375 | 11q23.3 | MLL | 117,860,137 | 117,860,239 | 1 | 4p12 | Q6AI58_HUMAN | 48,224,390 | 48,225,112 | 1 |
| DQ451147 | 264 | | 261 | 12p13.2 | BCL2L14 | 11,914,050 | 12,159,528 | 0 | 21q22.12 | RUNX1 | 35,181,194 | 35,187,132 | 1 |
| AJ130894 | 3,077 | | 2,199 | 13q13.3 | FAM48A | 36,481,631 | 36,531,787 | 1 | 2q35 | DES | 219,999,350 | 219,999,697 | 1 |
| AJ417079 | 296 | | 294 | 15q24.1 | PML_HUMAN | 72,112,550 | 72,112,669 | 1 | 17q21.2 | RARA | 35,752,582 | 35,758,217 | 1 |
| AJ420459 | 2,518 | | 1,290 | 12q24.11 | ATPBD1C | 109,374,761 | 109,390,423 | -1 | 9q34.11 | PHYHD1 | 130,729,194 | 130,744,502 | 1 |
| AJ420524 | 3,789 | | 735 | 16p13.3 | TNFRSF12A | 3,010,382 | 3,012,385 | 1 | 6q25.1 | LRP11 | 150,181,625 | 150,226,347 | 0 |
| AJ420584 | 1,923 | | 1,014 | 16q12.2 | AYTL1 | 54,123,325 | 54,175,082 | 1 | 6p24.3 | MUTED | 7,959,213 | 7,959,529 | 1 |
| AJ535465 | 376 | | 126 | 6p21.1 | TFEB | 41,767,193 | 41,767,302 | 1 | 11q13.1 | NR_002819.1 | 65,023,478 | 65,023,744 | 1 |
| AJ549093 | 1,270 | | 1,188 | 16p11.2 | FUS | 31,103,045 | 31,103,859 | 1 | 7q33 | CREB3L2 | 137,215,685 | 137,243,630 | 1 |
| AJ549094 | 1,270 | | 1,188 | 16p11.2 | FUS | 31,103,045 | 31,103,885 | 1 | 7q33 | CREB3L2 | 137,215,685 | 137,243,639 | 1 |
| Z35296 | 276 | | 186 | 21q22.12 | RUNX1 | 35,153,641 | 35,153,747 | 1 | 8q21.3 | MTG8_HUMAN | 93,098,677 | 93,098,835 | 1 |
| X06409 | 2,602 | | 1,677 | 11p15.4 | PRKCDBP | 6,297,199 | 6,298,295 | 1 | 3p25.2 | RAF1 | 12,600,107 | 12,616,916 | 0 |
| X81832 | 2,181 | | 1,473 | 12p12.1 | IAPP | 21,423,300 | 21,423,686 | 0 | 19q13.32 | GIPR | 50,863,354 | 50,877,253 | 0 |
| X98248 | 4,975 | | 2,553 | 1p13.3 | SORT1 | 109,657,624 | 109,742,096 | 1 | 6p21.1 | KLHDC3 | 43,089,996 | 43,096,987 | 1 |
| X74837 | 3,250 | | 1,875 | 9p21.2 | TEK | 27,150,653 | 27,151,403 | -1 | 6q22.31 | MAN1A1 | 119,542,016 | 119,711,788 | 0 |
| Z14955 | 1,073 | | 864 | 4q27 | IL2 | 123,594,315 | 123,597,015 | 1 | 16p13.13 | TNFRSF17 | 11,966,690 | 11,969,410 | 0 |
| X61177 | 2,024 | | 1,290 | 3p26.2 | IL5RA | 3,091,466 | 3,125,333 | 0 | 11q13.5 | C11orf67 | 77,265,302 | 77,266,043 | -1 |
| X60787 | 3,043 | | 1,629 | 20q13.32 | RAB22A | 56,368,139 | 56,368,545 | -1 | 17q25.3 | FOXK2 | 78,071,413 | 78,153,551 | 0 |
| AL137623 | 624 | | 429 | 9q22.31 | SUSD3 | 94,880,067 | 94,886,899 | 1 | 6 | IG | 17,693,452 | 17,693,949 | 0 |
| AL157434 | 3,588 | | 1,407 | 20q12 | PTPRT | 40,523,365 | 40,524,038 | 1 | 12q24.31 | ZCCHC8 | 121,523,388 | 121,549,822 | 1 |
| AL162013 | 1,758 | | 531 | 3q21.2 | PLXNA1 | 128,231,871 | 128,236,264 | 1 | 19q13.33 | SHANK1 | 55,854,335 | 55,854,829 | 1 |
| BX538323 | 2,445 | | 489 | 17p13.1 | SLC16A13 | 6,880,140 | 6,881,107 | 1 | 22q13.1 | JOSD1 | 37,411,568 | 37,413,018 | 1 |
| BX648452 | 2,464 | | 330 | 14q24.3 | LTBP2 | 74,045,389 | 74,046,589 | 1 | 17q23.3 | CYB561 | 58,863,399 | 58,865,533 | 1 |
| BX648622 | 4,052 | | 1,548 | 1p35.1 | HDAC1 | 32,530,299 | 32,571,814 | -1 | 12p13.31 | A2M | 9,111,575 | 9,133,849 | 1 |
| BX648647 | 4,845 | | 321 | 3q26.31 | NAALADL2 | 176,435,899 | 176,440,629 | 1 | 1 | IG | 558,777 | 559,341 | 0 |
| Z22957 | 4,281 | | 2,436 | 15q21.2 | MYO5A | 50,409,929 | 50,462,680 | 1 | 14q24.3 | NRXN3 | 79,208,347 | 79,210,162 | -1 |
| X86400 | 1,155 | | 378 | 11q23.3 | NA | 117,196,000 | 117,200,644 | 0 | 2q31.1 | NP_742067.2 | 170,602,941 | 170,603,500 | 1 |
| Y10351 | 2,956 | | 2,133 | 14q21.1 | PNN | 38,714,251 | 38,721,178 | 1 | 15q15.3 | SERF2_HUMAN | 41,872,465 | 41,873,488 | 1 |
| X66358 | 1,363 | | 1,074 | 17p13.3 | NP_057164.2 | 626,951 | 632,252 | -1 | 14q21.3 | CDKL1 | 49,866,470 | 49,932,367 | 0 |
| X77754 | 2,406 | | 969 | 11q13.3 | CCND1 | 69,167,877 | 69,175,457 | 1 | 1p32.2 | TACSTD2 | 58,813,689 | 58,815,485 | 1 |
| U02308 | 3,517 | | 2,067 | 2q36.1 | PAX3 | 222,793,103 | 222,867,267 | 0 | 13q14.11 | FOXO1A | 40,029,736 | 40,033,000 | 1 |
| U04847 | 1,857 | | 1,155 | 22q11.23 | SMARCB1 | 22,459,288 | 22,506,700 | 0 | 3p25.1 | ANKRD28 | 15,686,428 | 15,686,726 | -1 |
| U09850 | 3,908 | | 1,878 | 11p15.4 | ZNF143 | 9,449,437 | 9,506,104 | 1 | 14q24.3 | NP_569736.1 | 74,964,232 | 75,006,909 | -1 |
| U15426 | 942 | | 516 | 16 | IG | 8,855,468 | 8,855,754 | 0 | 12q13.12 | LASS5 | 48,815,821 | 48,824,108 | 1 |
| U22961 | 3,239 | | 1,365 | 12q13.12 | GPD1 | 48,786,318 | 48,788,032 | 1 | 4q13.3 | ALB | 74,488,882 | 74,502,189 | 1 |
| U28424 | 2,205 | | 1,512 | 11 | IG | 47,444,708 | 47,445,377 | 0 | 13q32.1 | DNAJC3 | 95,127,484 | 95,241,285 | 0 |
| U34343 | 681 | | 336 | 1p36.33 | MMP23B | 1,591,215 | 1,591,380 | 0 | 12q22 | NDUFA12 | 93,889,243 | 93,921,547 | 1 |
| U34919 | 2,745 | | 1,914 | 15q25.1 | ACSBG1 | 76,308,617 | 76,308,963 | 1 | 21q22.3 | ABCG1 | 42,518,904 | 42,590,020 | 1 |
| U39196 | 3,181 | | 1,503 | 5 | IG | 36,908,176 | 36,909,066 | 0 | 2q24.1 | KCNJ3 | 155,263,057 | 155,420,382 | 1 |
| U39402 | 2,210 | | 1,101 | 8q24.13 | MTSS1 | 125,633,162 | 125,633,809 | -1 | 5q33.1 | NP_060517.1 | 150,050,842 | 150,056,458 | 1 |
| U60666 | 2,446 | | 1,389 | 7p14.1 | GLI3 | 42,146,589 | 42,147,342 | -1 | 8q24.22 | LRRC6 | 133,653,532 | 133,756,998 | 1 |
| U66406 | 3,394 | | 1,020 | 8 | IG | 43,097,759 | 43,098,151 | 0 | 17p13.1 | EFNB3 | 7,549,429 | 7,555,370 | 1 |
| U79248 | 1,615 | | 195 | 12 | IG | 52,915,715 | 52,916,050 | 0 | 18 | IG | 5,233,923 | 5,235,189 | 0 |
| U80737 | 3,487 | | 978 | Xq23 | DCX | 110,426,037 | 110,426,656 | 1 | 20q13.12 | NCOA3 | 45,709,222 | 45,717,346 | 1 |
| U80747 | 2,013 | | 837 | 4q31.1 | MAML3 | 141,029,529 | 141,031,160 | 1 | 2q36.1 | SERPINE2 | 224,548,238 | 224,548,634 | -1 |
| U82811 | 747 | | 591 | 3p14.3 | HESX1 | 57,207,260 | 57,209,023 | 1 | 11q14.3 | Q96AU6_HUMAN | 91,808,635 | 91,808,777 | -1 |
| U87460 | 4,156 | | 1,839 | 9 | IG | 122,560,812 | 122,562,549 | 0 | 7q31.33 | GPR37 | 124,173,294 | 124,192,749 | 1 |
| U94364 | 3,184 | | 1,383 | 11q12.2 | PRPF19 | 60,414,772 | 60,415,085 | -1 | Xp22.33 | GYG2 | 2,758,146 | 2,810,583 | 1 |
| X83127 | 1,624 | | 1,203 | 3q25.31 | KCAB1_HUMAN | 157,492,289 | 157,737,358 | 1 | 2p11.2 | TCF7L1 | 85,291,258 | 85,291,388 | 1 |
| X99270 | 2,000 | | 885 | 6p25.3 | GMDS | 1,569,040 | 1,570,735 | -1 | Xq28 | TREX2 | 152,366,320 | 152,383,690 | 1 |
| L34409 | 1,093 | | 243 | 4p16.3 | NOP14_HUMAN | 2,929,886 | 2,930,609 | -1 | 16 | IG | 7,181,414 | 7,181,780 | 0 |
| M63394 | 1,354 | | 591 | 22q12.3 | TIMP3 | 31,588,454 | 31,589,028 | 0 | 1q21.3 | NA | 151,500,481 | 151,501,222 | 0 |
| D15050 | 5,316 | | 3,375 | 10p11.22 | TCF8 | 31,648,167 | 31,856,314 | 0 | 21q22.3 | SN1L1_HUMAN | 43,658,829 | 43,660,658 | 1 |
| L34058 | 2,690 | | 2,139 | 22 | IG | 25,400,342 | 25,400,719 | 0 | 16q23.3 | CDH13 | 81,218,134 | 82,386,238 | 0 |
| L22569 | 2,286 | | 459 | 8p23.1 | CTSB | 11,739,247 | 11,742,716 | 1 | 3p22.1 | NKTR | 42,664,222 | 42,665,199 | -1 |
| M73792 | 1,538 | | 207 | 2q21.3 | RAB3GAP1 | 135,643,108 | 135,644,326 | -1 | 10p15.1 | CALML3 | 5,557,922 | 5,558,226 | 1 |
| M30773 | 2,548 | | 510 | 20q11.22 | ERGIC3 | 33,607,410 | 33,608,749 | -1 | 2p14 | PPP3R1 | 68,260,322 | 68,333,120 | 1 |
| M14219 | 1,778 | | 1,077 | 12q21.33 | PGS2_HUMAN | 90,063,835 | 90,100,585 | 1 | 6p24.3 | SSR1 | 7,233,052 | 7,233,538 | 1 |
| M95586 | 4,410 | | 1,722 | 19p13.3 | TCF3 | 1,570,109 | 1,601,277 | 1 | 17q22 | HLF | 50,753,021 | 50,755,891 | 1 |
| J05016 | 2,865 | | 1,980 | 7q36.1 | PDIA4 | 148,331,450 | 148,356,477 | 1 | 10q11.21 | Mar-08 | 45,270,047 | 45,270,524 | 1 |
| M90820 | 1,815 | | 669 | 14q21.2 | FKBP3 | 44,654,886 | 44,673,415 | 0 | 19p13.3 | PIP5K1C | 3,581,214 | 3,582,072 | 0 |
| M90309 | 1,513 | | 1,017 | 20p11.23 | HARS2 | 18,692,002 | 18,692,527 | 1 | 14q21.2 | FKBP3 | 44,654,855 | 44,673,414 | 0 |
| M12996 | 2,446 | | 1,086 | Xq28 | G6PD | 153,412,821 | 153,415,905 | 1 | 17q24.2 | PRKCA | 62,236,528 | 62,237,303 | 1 |
| D28480 | 2,327 | | 1,629 | 16p13.13 | Q8N2X2_HUMAN | 12,335,894 | 12,336,243 | 1 | 7q22.1 | MCM7 | 99,528,340 | 99,534,830 | 0 |
| D38081 | 2,932 | | 1,029 | 17q23.3 | ERN1 | 59,503,337 | 59,504,041 | -1 | 19p13.3 | TBXA2R | 3,545,777 | 3,557,730 | 0 |
| M94654 | 3,465 | | 1,491 | 20q13.32 | RAB22A | 56,368,139 | 56,368,545 | -1 | 17q25.3 | FOXK2 | 78,071,413 | 78,153,551 | 0 |
| M35718 | 3,025 | | 2,103 | 10q26.12 | FGFR2_HUMAN | 123,233,222 | 123,347,902 | 1 | 1q22 | C1orf43 | 152,446,336 | 152,446,725 | -1 |
| J04168 | 2,288 | | 1,293 | 16p11.2 | SPN | 29,582,080 | 29,584,328 | 1 | 8q12.3 | YTHDF3 | 64,262,739 | 64,263,150 | -1 |
| M19695 | 561 | | 255 | 22q11.23 | BCR | 21,961,704 | 21,962,601 | 1 | 9q34.12 | ABL1 | 132,645,577 | 132,645,940 | 1 |
| L22179 | 6,940 | | 6,936 | 11q23.3 | MLL | 117,812,435 | 117,860,239 | 0 | 4q21.3 | AFF1 | 88,224,288 | 88,275,877 | 1 |
| D38522 | 5,974 | | 1,293 | 1q22 | SYT11 | 154,095,955 | 154,120,685 | 1 | 13q12.3 | HMGB1 | 30,006,774 | 30,008,473 | 1 |
| M76729 | 7,138 | | 5,514 | 9q34.3 | COL5A1 | 136,673,626 | 136,874,560 | 0 | 11q12.3 | NP_955369.1 | 62,311,463 | 62,314,294 | -1 |
| L11370 | 4,069 | | 3,195 | 8p21.1 | CCDC25 | 27,670,301 | 27,670,756 | 1 | 5q31.3 | PCDH1 | 141,222,402 | 141,229,181 | 1 |
| L11372 | 918 | | 327 | 5q31.3 | PCDHGA12 | 140,837,590 | 140,837,913 | 1 | 8q24.12 | NP_076999.1 | 120,915,486 | 120,916,085 | -1 |
| M95929 | 1,433 | | 597 | 1q24.3 | PRRX1 | 168,900,038 | 168,972,273 | 1 | 10q24.31 | SFXN3 | 102,790,626 | 102,790,919 | 1 |
| M55580 | 978 | | 516 | Xp22.11 | SAT | 23,711,385 | 23,714,044 | 1 | 10 | IG | 75,215,716 | 75,218,875 | 0 |
| M26658 | 2,490 | | 2,082 | 1p36.13 | CAPZB | 19,538,441 | 19,543,510 | 1 | 17q23.3 | ACE | 58,916,423 | 58,928,711 | 1 |
| M11722 | 2,068 | | 1,851 | 2q32.1 | DNAJC10 | 183,332,201 | 183,335,773 | 1 | 10q24.1 | DNTT | 98,054,156 | 98,088,185 | 1 |
| M19267 | 1,633 | | 852 | 8q12.1 | CHCHD7 | 57,289,709 | 57,291,643 | -1 | 15q22.2 | TPM1 | 61,122,014 | 61,150,911 | 0 |
| M86699 | 3,866 | | 2,571 | 3p21.1 | DCP1A | 53,309,409 | 53,310,337 | -1 | 6q14.1 | TTK | 80,772,269 | 80,808,952 | 1 |
| AF085869 | 567 | | 231 | 2p25.1 | ROCK2 | 11,331,694 | 11,331,966 | 1 | 4 | IG | 71,951,265 | 71,951,566 | 0 |
| AF086522 | 484 | | 135 | 5q12.3 | ERBB2IP | 65,281,447 | 65,281,579 | 1 | 10q22.2 | SYNPO2L | 75,075,282 | 75,075,638 | 1 |
| AL365412 | 1,001 | | 432 | 17q12 | LASP1 | 34,324,104 | 34,324,214 | 1 | 19p13.3 | PLAC2 | 5,509,182 | 5,510,039 | 1 |
| AL355736 | 1,603 | | 315 | 15q24.3 | HMG20A | 75,500,332 | 75,545,502 | 0 | 11p12 | TTC17 | 43,361,774 | 43,362,286 | 1 |
| AL355682 | 887 | | 159 | 6q21 | ATG5 | 106,766,299 | 106,766,792 | 1 | 21q22.3 | UBE2G2 | 45,013,383 | 45,013,761 | 1 |
| S48220 | 2,222 | | 459 | 1 | IG | 54,132,466 | 54,149,347 | 0 | 12q24.32 | NA | 126,216,501 | 126,216,940 | 1 |
| U95646 | 1,320 | | 486 | 1 | IG | 554,939 | 555,517 | 0 | 15q22.31 | PPIB | 62,235,069 | 62,241,403 | 0 |
| AL050025 | 4,012 | | 1,566 | 16q22.2 | AP1G1 | 70,333,686 | 70,362,603 | 1 | 15q22.2 | CA12 | 61,402,786 | 61,403,978 | 1 |
| AL049378 | 1,769 | | 450 | 5q13.2 | TNPO1 | 72,244,896 | 72,245,971 | 1 | 19p13.3 | PTPRS | 5,202,441 | 5,203,106 | 1 |
| AL122070 | 2,597 | | 702 | 4p13 | NP_001025126.1 | 41,679,052 | 41,681,246 | 1 | 5q35.1 | NA | 172,314,391 | 172,314,789 | 0 |
| AL832738 | 5,325 | | 1,542 | 22q11.23 | GGT1 | 23,329,114 | 23,354,101 | 1 | 6 | IG | 2,886,136 | 2,889,264 | 0 |
| AL833498 | 2,003 | | 561 | 2 | IG | 208,206,816 | 208,207,495 | 0 | 16 | IG | 73,038,839 | 73,040,132 | 0 |
| AL833879 | 5,242 | | 2,175 | 19q13.42 | LENG8 | 59,657,441 | 59,664,392 | 1 | 15q15.1 | NP_055953.1 | 39,496,767 | 39,544,296 | 0 |
| BX537721 | 10,009 | | 4,188 | 5q14.1 | CMYA5 | 79,067,956 | 79,131,797 | -1 | 14 | IG | 56,827,426 | 56,832,198 | 0 |
| BX537739 | 2,533 | | 276 | 6p21.1 | PARC_HUMAN | 43,257,949 | 43,258,445 | 1 | 4q13.3 | MUC7 | 71,381,277 | 71,383,303 | 1 |
| BX537789 | 3,024 | | 318 | 8p21.3 | PIWIL2 | 22,231,675 | 22,233,199 | 1 | 2p14 | VPS54 | 64,078,188 | 64,079,679 | 1 |
| BX538125 | 1,416 | | 243 | 10q23.2 | SNCG | 88,710,762 | 88,711,878 | 1 | 1 | IG | 559,334 | 559,619 | 0 |
| BX640810 | 3,020 | | 315 | 2q34 | FYV1_HUMAN | 208,892,354 | 208,894,998 | 1 | 4q13.3 | SMR3B | 71,290,186 | 71,290,550 | 1 |
| BX640830 | 2,726 | | 546 | 10 | IG | 10,701,478 | 10,702,908 | 0 | 8 | IG | 38,070,953 | 38,072,184 | 0 |
| BX640852 | 4,838 | | 738 | 7q31.32 | WASL | 123,108,806 | 123,111,202 | -1 | 6p21.1 | RL7L_HUMAN | 42,955,632 | 42,965,558 | 1 |
| BX640894 | 3,936 | | 396 | X | IG | 71,445,452 | 71,446,734 | 0 | 4p16.3 | PCGF3 | 751,289 | 753,931 | 1 |
| BX647356 | 4,328 | | 1,725 | 12q13.2 | SUOX | 54,677,321 | 54,685,337 | -1 | 19p13.2 | RAB11B | 8,361,283 | 8,375,322 | 0 |
| BX647833 | 3,768 | | 753 | 1p13.2 | AMPD1 | 115,028,354 | 115,039,693 | 1 | 3q29 | FYTTD1 | 198,967,792 | 198,995,564 | 1 |
| BX648432 | 6,046 | | 540 | 19q13.11 | KIAA0355 | 39,437,331 | 39,538,311 | 0 | 3q26.31 | PLD1 | 172,801,323 | 172,820,930 | 1 |
| BX648752 | 3,008 | | 513 | 6 | IG | 30,040,832 | 30,046,456 | 0 | 11p15.5 | BET1L | 192,924 | 195,980 | 1 |
| BX648852 | 3,390 | | 303 | 21q22.11 | ITSN1 | 34,015,412 | 34,036,947 | 1 | 1 | IG | 554,327 | 554,676 | 0 |
| AF109189 | 2,325 | | 801 | Xq26.3 | Q96HG1_HUMAN | 133,952,723 | 133,953,632 | 1 | 12p13.31 | A2M | 9,111,571 | 9,121,685 | 1 |
| AF113676 | 2,571 | | 1,254 | 15q25.3 | AKAP13 | 83,878,417 | 83,879,486 | -1 | 14q32.13 | SERPINA1 | 93,914,451 | 93,924,693 | 1 |
| AF113680 | 2,378 | | 1,092 | 6p21.2 | KIF6 | 39,656,262 | 39,656,920 | -1 | 9q22.31 | IARS | 94,012,448 | 94,052,033 | 1 |
| AF113700 | 1,286 | | 501 | 1q21.3 | SELENBP1 | 149,603,418 | 149,605,458 | -1 | 14q23.3 | CHURC1 | 64,450,929 | 64,468,782 | 1 |
| AF116614 | 1,523 | | 1,038 | 16p12.1 | POLR3E | 22,239,477 | 22,239,686 | -1 | 20q11.22 | ERGIC3 | 33,593,291 | 33,608,819 | 1 |
| AF116626 | 1,326 | | 306 | 11p15.5 | H19 | 1,975,379 | 1,975,557 | -1 | 3q26.31 | Q9P1K3_HUMAN | 173,843,805 | 173,844,937 | 0 |
| AF116645 | 1,251 | | 849 | 3q22.1 | TF | 134,973,360 | 134,973,599 | -1 | 4q13.3 | ALB | 74,498,153 | 74,505,823 | 1 |
| AF116693 | 1,299 | | 243 | 6p21.2 | ZFAND3 | 38,068,317 | 38,068,463 | -1 | 8p21.2 | SLC25A37 | 23,483,881 | 23,485,040 | 1 |
| AF118076 | 2,114 | | 525 | 12p12.2 | PDE3A | 20,488,534 | 20,489,227 | -1 | 1q41 | CENPF | 212,888,646 | 212,894,385 | 1 |
| AF118081 | 1,374 | | 378 | 11p15.5 | H19 | 1,975,061 | 1,975,611 | -1 | 19q13.12 | NP_775907.3 | 41,252,983 | 41,253,812 | 1 |
| AF118090 | 1,759 | | 771 | 19q12 | ZNF536 | 35,555,468 | 35,556,172 | -1 | 4q13.3 | ALB | 74,498,177 | 74,505,837 | 1 |
| AF118092 | 1,826 | | 1,311 | 4q31.3 | FGG | 155,744,738 | 155,753,253 | -1 | 5p13.1 | OSMR | 38,971,303 | 38,971,500 | 1 |
| AF119840 | 2,720 | | 1,827 | 12p12.1 | CMAS | 22,106,569 | 22,107,219 | -1 | 4q13.3 | ALB | 74,488,868 | 74,505,837 | 1 |
| AF119846 | 1,807 | | 705 | 6q25.1 | LATS1 | 150,047,212 | 150,080,900 | -1 | 19q13.2 | EIF3S12 | 43,801,714 | 43,819,432 | 0 |
| AF119870 | 2,211 | | 366 | 10q21.3 | CTNNA3 | 68,583,800 | 68,584,897 | -1 | 6q15 | C6orf166 | 88,466,746 | 88,467,863 | -1 |
| AF119879 | 1,468 | | 264 | 15q13.1 | Q9P168_HUMAN | 27,872,347 | 27,873,443 | 0 | 5q14.1 | PAPD4 | 79,001,915 | 79,002,244 | -1 |
| AF130074 | 1,741 | | 351 | 5p12 | HMGCS1 | 43,337,314 | 43,338,321 | 0 | 9q34.11 | C9orf32 | 131,435,038 | 131,435,776 | 1 |
| AF130098 | 1,668 | | 612 | 2q37.1 | SPP2 | 234,635,425 | 234,636,524 | -1 | 11 | IG | 5,226,079 | 5,232,556 | 0 |
| AF130109 | 1,517 | | 894 | 19q13.2 | TIM50_HUMAN | 44,664,455 | 44,672,404 | -1 | 20q13.33 | PSMA7 | 60,145,184 | 60,147,639 | 1 |
| AF130111 | 1,604 | | 669 | 11q13.1 | NR_002819.1 | 65,025,161 | 65,025,468 | -1 | 5q31.3 | HDAC3 | 140,980,632 | 140,988,401 | 1 |
| AF130113 | 1,874 | | 765 | 16q22.1 | CYB5B | 68,016,008 | 68,054,245 | -1 | Xp11.21 | ALAS2 | 55,052,216 | 55,060,772 | 1 |
| AF208859 | 2,764 | | 1,026 | Xq22.1 | ARMCX3 | 100,764,818 | 100,768,447 | 1 | 21 | IG | 26,754,093 | 26,754,534 | 0 |
| AF289572 | 1,334 | | 438 | 1q44 | HNRPU | 243,081,044 | 243,081,234 | 1 | 16p13.3 | C16orf33 | 44,960 | 47,444 | 1 |
| AF289585 | 2,310 | | 924 | 15q14 | C15orf29 | 32,282,823 | 32,283,129 | 1 | 3q29 | FAM43A | 195,888,565 | 195,890,561 | 1 |
| AF289607 | 1,334 | | 438 | 1q44 | HNRPU | 243,081,044 | 243,081,234 | 1 | 16p13.3 | C16orf33 | 44,960 | 47,444 | 1 |
| AF318349 | 4,715 | | 1,236 | 6p21.31 | NP_851853.1 | 33,846,968 | 33,864,760 | -1 | 17q12 | ERBB2 | 35,134,887 | 35,138,437 | 1 |
| AF333762 | 761 | | 303 | 19q13.42 | Q9BZ77_HUMAN | 60,910,683 | 60,911,143 | 1 | 16 | IG | 31,726,406 | 31,726,705 | 0 |
| AF351612 | 3,420 | | 501 | 3q11.2 | NSUN3 | 95,264,524 | 95,286,913 | 1 | 6q25.3 | VIL2 | 159,139,670 | 159,141,556 | 1 |
| AF363068 | 566 | | 282 | 18 | IG | 19,969,430 | 19,969,791 | 0 | 20q13.12 | NCOA3 | 45,574,151 | 45,574,363 | -1 |
| AF370417 | 4,557 | | 756 | 22q11.23 | MMP11 | 22,445,044 | 22,456,845 | 0 | 7p13 | YKT6 | 44,217,079 | 44,220,416 | 1 |
| AF370429 | 2,929 | | 918 | 1q21.1 | PDE4DIP | 143,387,794 | 143,751,128 | 0 | 19p13.3 | HM20B_HUMAN | 3,523,974 | 3,530,081 | 0 |
| AF447875 | 4,952 | | 450 | 22 | IG | 26,575,385 | 26,577,447 | 0 | 14q32.2 | Q96NA9_HUMAN | 100,369,700 | 100,397,116 | 0 |
| BC001160 | 2,229 | | 639 | 9q34.3 | LHX3 | 138,228,286 | 138,228,726 | 1 | 11p11.2 | DDB2 | 47,193,147 | 47,217,344 | 1 |
| BC004486 | 4,179 | | 963 | 22q12.1 | NP_775781.1 | 27,498,719 | 27,513,164 | 1 | 17q11.2 | BLMH | 25,599,347 | 25,643,125 | 0 |
| BC007843 | 3,689 | | 1,485 | 12p13.31 | COPS7A | 6,703,483 | 6,711,302 | -1 | 8q24.3 | GRINA | 145,136,264 | 145,139,570 | 1 |
| BC008476 | 1,210 | | 231 | 13q21.32 | PCDH9 | 66,646,433 | 66,647,457 | 1 | 12q15 | CNOT2 | 68,963,533 | 68,963,722 | 1 |
| BC013363 | 3,008 | | 1,473 | 7q34 | Q9H0M3_HUMAN | 138,252,978 | 138,254,676 | -1 | 13q12.2 | GTF3A | 26,896,782 | 26,907,827 | 0 |
| BC014141 | 3,154 | | 1,425 | 12q15 | SLC35E3 | 67,426,259 | 67,445,018 | 1 | 16q13 | KATNB1 | 56,342,300 | 56,348,657 | 1 |
| BC015906 | 1,652 | | 384 | 17q21.32 | ITGB3 | 42,744,698 | 42,745,030 | 1 | 15q13.3 | Q6ZSY1_HUMAN | 30,849,377 | 30,850,684 | 1 |
| BC018851 | 2,145 | | 726 | 17q25.1 | KIAA0195 | 71,007,057 | 71,007,764 | -1 | 19q13.32 | PRKD2 | 51,869,413 | 51,887,076 | 0 |
| BC019054 | 2,856 | | 1,338 | 20p13 | SMOX | 4,106,151 | 4,116,367 | -1 | 9q34.11 | C9orf74 | 130,173,461 | 130,192,834 | 1 |
| BC020349 | 1,003 | | 213 | 17q21.31 | CG69_HUMAN | 39,752,926 | 39,753,119 | -1 | 12q13.13 | HOXC6 | 52,696,959 | 52,709,740 | 1 |
| BC020793 | 1,461 | | 243 | 1q42.3 | LYST | 234,059,331 | 234,096,798 | 0 | 2p11.2 | SFTPB | 85,738,889 | 85,739,187 | 1 |
| BC022263 | 1,066 | | 570 | 10q11.23 | ACF_HUMAN | 52,265,994 | 52,315,391 | 1 | 15q22.31 | ANKDD1A | 63,032,080 | 63,032,552 | -1 |
| BC023515 | 4,676 | | 1,818 | 5q34 | SLIT3 | 168,130,900 | 168,204,434 | 0 | Xp11.21 | MAGED2 | 54,851,559 | 54,859,168 | 1 |
| BC024102 | 3,167 | | 822 | 8q24.3 | TIGD5 | 144,751,909 | 144,753,362 | -1 | 1p36.33 | NA | 556,222 | 557,840 | -1 |
| BC029403 | 982 | | 276 | 12q23.1 | SLC25A3 | 97,511,599 | 97,511,943 | 1 | 5 | IG | 6,544,878 | 6,545,706 | 0 |
| BC035171 | 3,798 | | 1,335 | 4 | IG | 140,743,287 | 140,747,303 | 0 | 7p14.3 | KBTB2_HUMAN | 32,874,313 | 32,886,080 | 0 |
| BC035765 | 1,690 | | 387 | 16p13.12 | NP_060810.1 | 12,760,640 | 12,762,520 | -1 | 14q12 | NP_542395.1 | 30,985,947 | 30,986,674 | 1 |
| BC036586 | 1,058 | | 183 | 8q12.1 | XKR4 | 56,386,450 | 56,386,963 | 1 | 1 | IG | 559,094 | 559,616 | 0 |
| BC039466 | 1,401 | | 393 | 21q22.3 | SLC19A1 | 45,755,527 | 45,756,929 | 0 | 20q11.22 | ITGB4BP | 33,330,139 | 33,335,769 | 1 |
| BC071903 | 2,427 | | 1,644 | 19q13.42 | ZNF347 | 58,343,988 | 58,354,106 | -1 | 22q12.3 | EIF3S7 | 35,236,849 | 35,252,127 | 1 |
